# Cholesterol-dependent dynamic changes in the conformation of the type 1 cholecystokinin receptor affect ligand binding and G protein coupling

**DOI:** 10.1371/journal.pbio.3002673

**Published:** 2024-07-31

**Authors:** Kaleeckal G. Harikumar, Peishen Zhao, Brian P. Cary, Xiaomeng Xu, Aditya J. Desai, Maoqing Dong, Jesse I. Mobbs, Chirine Toufaily, Sebastian G. B. Furness, Arthur Christopoulos, Matthew J. Belousoff, Denise Wootten, Patrick M. Sexton, Laurence J. Miller

**Affiliations:** 1 Department of Molecular Pharmacology and Experimental Therapeutics, Mayo Clinic, Scottsdale, Arizona, United States of America; 2 Drug Discovery Biology Theme, Monash Institute of Pharmaceutical Sciences, Monash University, Parkville, Australia; 3 ARC Centre for Cryo-electron Microscopy of Membrane Proteins, Monash Institute of Pharmaceutical Sciences, Monash University, Parkville, Australia; 4 School of Biomedical Sciences, University Queensland, Queensland, Australia; National Cancer Institute, UNITED STATES

## Abstract

Development of optimal therapeutics for disease states that can be associated with increased membrane cholesterol requires better molecular understanding of lipid modulation of the drug target. Type 1 cholecystokinin receptor (CCK1R) agonist actions are affected by increased membrane cholesterol, enhancing ligand binding and reducing calcium signaling, while agonist actions of the closely related CCK2R are not. In this work, we identified a set of chimeric human CCK1R/CCK2R mutations that exchange the cholesterol sensitivity of these 2 receptors, providing powerful tools when expressed in CHO and HEK-293 model cell lines to explore mechanisms. Static, low energy, high-resolution structures of the mutant CCK1R constructs, stabilized in complex with G protein, were not substantially different, suggesting that alterations to receptor dynamics were key to altered function. We reveal that cholesterol-dependent dynamic changes in the conformation of the helical bundle of CCK receptors affects both ligand binding at the extracellular surface and G protein coupling at the cytosolic surface, as well as their interrelationships involved in stimulus-response coupling. This provides an ideal setting for potential allosteric modulators to correct the negative impact of membrane cholesterol on CCK1R.

## Abstract 2

A previous study in PLOS Biology solved the cryo-EM structure of the cholecystokinin type 1 receptor (CCK1R) to gain insights into the mechanisms governing G protein selectivity. This Update Article provides a structural and biophysical characterization of the effects of cholesterol on ligand binding and G protein coupling at the receptor.

## Introduction

Development of receptor-targeting drugs has often utilized high-throughput assays in model systems with receptors overexpressed in generic membranes/cells. However, it is now appreciated that many G protein-coupled receptors directly associate with cholesterol in the membrane bilayer, with this interaction capable of affecting the function of these receptors [1–4]. It is notable that the disease states for which some of these receptors may be therapeutically targeted can be associated with abnormal lipid composition [3,5,6], yet this has often been overlooked in developing or validating those therapeutics. The type 1 cholecystokinin receptor (CCK1R) is an example of this scenario, where the receptor is a potential target for drug treatment of obese patients who can have excess cholesterol in their cell membranes [7–9]. Elevated membrane cholesterol occurs in some patients with obesity and metabolic syndrome, a group representing a prime target for such drugs [5,6]. Inadequate understanding of the impact of the lipid environment of CCK1R may have contributed to current lack of success in developing drugs targeting this receptor for management of obesity.

The CCK1R is also ideal for study of the molecular basis of the impact of membrane cholesterol on G protein-coupled receptors (GPCRs), since it is structurally highly homologous to CCK2R that is not modulated by changes in membrane cholesterol, but binds to and is activated by the same endogenous CCK-8 peptide with similar, predominant, Gq/11-dependent signaling [10,11]. Indeed, an unbiased approach, utilizing systematic domain exchange within chimeric CCK1R/CCK2R constructs to identify the region of CCK1R responsible for its cholesterol sensitivity, demonstrated that a cholesterol-association (CRAC) motif ((L/V)-X_1-5_-Y-X_1-5_-(R/K)) in transmembrane segment 3 (TM3) of CCK1R was responsible for the functional impact of changes in cholesterol levels [10]. This was true despite the presence of multiple cholesterol-association motifs distributed throughout both CCK receptors [11], and multiple cholesterols that are observed in structures of both CCK receptors [12,13]. This TM3 CRAC motif is the site of direct interaction with cholesterol that mediates the aberrant CCK-8 stimulus-activity coupling observed in vitro in membranes with increased levels of this lipid [10]. This effect has clear structure-activity dependency, with other sterols, such as the bile acid ursodeoxycholic acid [14] and the phytosterol β-sitosterol [15], also acting through this site to reverse the detrimental effect of cholesterol at CCK1R. The modulatory impact of cholesterol on this receptor has also been observed in vivo, in gallbladders of humans and animal models with cholesterol gallstones [16–19], as well as in obese patients without gallstones [20].

Disruption of this specific CCK1R CRAC motif by replacing Tyr140^3.51^ with Ala (Y140A) can eliminate the cholesterol sensitivity of this receptor [21] by mimicking the function of this receptor in elevated cholesterol, with this construct exhibiting increased binding affinity of CCK-8 and reduced CCK-8-stimulated intracellular calcium signaling [21]. Of note, CCK2R also has a CRAC motif in the same position, yet that receptor is not affected by excess cholesterol [21], and the analogous Ala mutation of Tyr153^3.51^ residue in CCK2R has no impact on CCK binding, biological activity, or cholesterol dependence [21].

In this work, we broadly characterized the CRAC motif Tyr residue, as well as surrounding residues, allowing us to identify a chimeric CCK1R/CCK2R construct that eliminated cholesterol sensitivity, while retaining other functional characteristics of wild-type (WT) CCK1R. Importantly, the opposite chimeric construct of CCK2R/CCK1R provided gain-of-function, introducing cholesterol sensitivity into CCK2R. These novel tools were utilized in high-resolution structural determination of CCK-receptor-G protein complexes using cryo-electron microscopy (cryo-EM) and in the analysis of dynamic events occurring at the extracellular face of the receptor involved in ligand recognition and at the cytosolic face of the receptor involved in G protein coupling. We pharmacologically characterized peptide structure-activity relationships for binding and activation, the kinetics of CCK association and dissociation, and its microenvironment using photoaffinity labeling and fluorescence studies. The latter were used to quantify G protein conformational changes and activation. These studies revealed dynamic interdependency between extracellular and intracellular events that contribute to the mechanism of cholesterol sensitivity of CCK1R and provide insights that could inform future development of drugs targeting this cholesterol-sensitive GPCR.

## Results/Discussion

### Structure-activity studies of the key CCK1R cholesterol-binding motif

**Fig 1:**
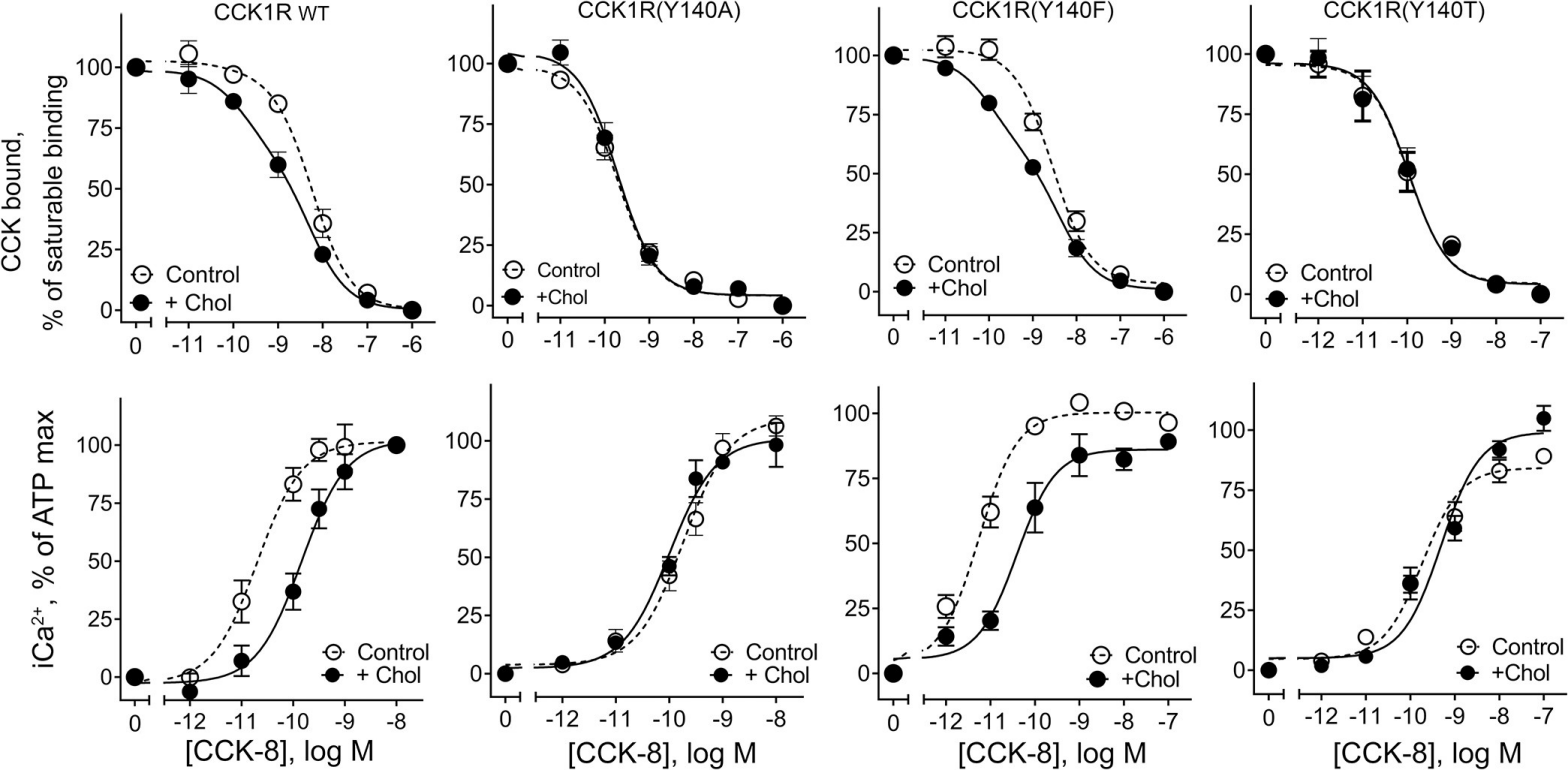
Characterization of CCK binding and biological activity at Tyr140^3.51^ variants. Shown are competition-binding curves (top row) and intracellular calcium responses (bottom row) of CCK1R Tyr140^3.51^ variants expressed in CHO-K1 cells in the absence or presence of enhanced membrane cholesterol. Binding values are expressed as percentages of saturable binding of CCK radioligand. Intracellular calcium responses are expressed as percentages of maximal responses to ATP (0.1 mM). The plots represent mean ± SEM of a minimum of 4 independent experiments performed in duplicate. Underlying data can be found in S1 Data.

Tyr140^3.51^ is a key residue in the CRAC motif, which is situated low in CCK1R TM3 and is critical for the functional impact of high cholesterol and other sterols on this receptor [11]. Disruption of this CRAC motif by replacing the Tyr with Ala (Y140A) eliminates cholesterol sensitivity of CCK1R, while mimicking the function of this receptor in a high cholesterol environment [21]. Here, we further explored the chemical requirements for cholesterol sensitivity through replacement of Tyr140^3.51^ with the non-hydroxylated aromatic residue, Phe (Y140F), or with Thr that contains a hydroxyl group, but lacks the ring structure of Tyr (Y140T). The functional characterization and impact of increased cholesterol on these constructs expressed in CHO-K1 cells is shown in Fig 1 and Table 1. The Y140F construct was sensitive to increased cholesterol, similar to WT CCK1R, with elevated membrane cholesterol producing a high affinity ligand-binding site not seen in the absence of elevated membrane cholesterol, while the low affinity binding site was unchanged (Fig 1, top row). This was also associated with reduced intracellular calcium response to CCK-8 (Fig 1, bottom row). In contrast, like CCK1R(Y140A), the Y140T mutation resulted in a 10-fold increase in CCK binding affinity of a predominant single site, and decreased intracellular calcium responses relative to WT CCK1R, with these parameters similar in normal or elevated membrane cholesterol.

**Table 1:** Binding affinity and biological activity of CCK-8 at CCK receptor constructs expressed in native and cholesterol-enriched HEK-293 cells or CHO-K1 cells (SRD15-CCK1R cells). Underlying data are found in S1 Data.

Constructs	pKi (“n”)	*P* values compared to control	B_max_x10^5^sites/cell	*P* values compared to control	iCa^2+^, pEC_50_ (“n”)	*P* values compared to control
***CHO-K1 cells***						
CCK1R WT Control + Chol	8.3±0.1 (4) 9.5±0.1 (high)*; 8.1±0.2 (low) (4)	0.05	0.6±0.1 0.6±0.1	0.886	10.8±0.1 (6) 9.9±0.1* (6)	0.002
CCK1R(Y140A) Control + Chol	9.7±0.1 (4) 9.7±0.1 (4)	0.886	0.5±0.1 0.5±0.1	>0.999	9.7±0.2 (5) 10.0±0.2 (5)	0.309
CCK1R(Y140F) Control + Chol	8.6±0.1 (5) 9.9±0.3 (high)*; 8.1±0.3 (low) (5)	0.008	0.6±0.1 0.7±0.1	0.222	11.2±0.1 (6) 10.3±0.1* (6)	0.002
CCK1R(Y140T) Control + Chol	10.3±0.2 (5) 10.3±0.2 (5)	0.968	0.5±0.1 0.3±0.1	0.309	9.7±0.2 (6) 9.3±0.2 (6)	0.132
***HEK-293 cells***						
CCK1R WT Control + Chol	8.9±0.1 (4) 10±0.2(high)*; 8.0±0.3 (low) (4)	0.029	0.7±0.1 0.7±0.1	0.486	11.1±0.2 (5) 10.2±0.1* (5)	0.008
CCK1R(sterol 7M) Control + Chol	8.7±0.2 (4) 8.8±0.2 (4)	>0.999	0.7±0.1 0.7±0.1	0.686	11.4±0.3 (5) 11.3±0.3 (5)	0.730
CCK2R WT Control + Chol	9.0±0.1 (4) 8.9±0.2 (4)	>0.999	0.6±0.1 0.7±0.1	0.686	10.2±0.2 (5) 10.5±0.2 (5)	0.421
CCK2R(sterol 7M) Control + Chol	8.8±0.1 (5) 9.3±0.1* (5)	0.008	0.6±0.1 0.7±0.1	0.691	10.2±0.2 (8) 9.4±0.1* (8)	0.010
***SRD-CCK1R cells***						
CHO-CCK1R SRD15-CCK1R WT	8.8±0.1 (4) 9.8±0.2 (high)*; 7.8±0.2 (low) (5)	0.016	0.6±0.1 0.7±0.1	0.730	11.0±0.1 (7) 9.8±0.1* (5)	0.003

Values are expressed as mean ± SEM from duplicate determinations in “n” independent experiments (replicate number in parentheses). Data were analyzed with unpaired *t* test with Mann–Whitney post-test.

* *p* < 0.05, significantly different from the control for the same cell line. Analogous data for the SRD15-CCK1R cell line with elevated cholesterol was previously published in Harikumar and colleagues, Lipids 48:231–244, 2013 [10].

**Fig 2:**
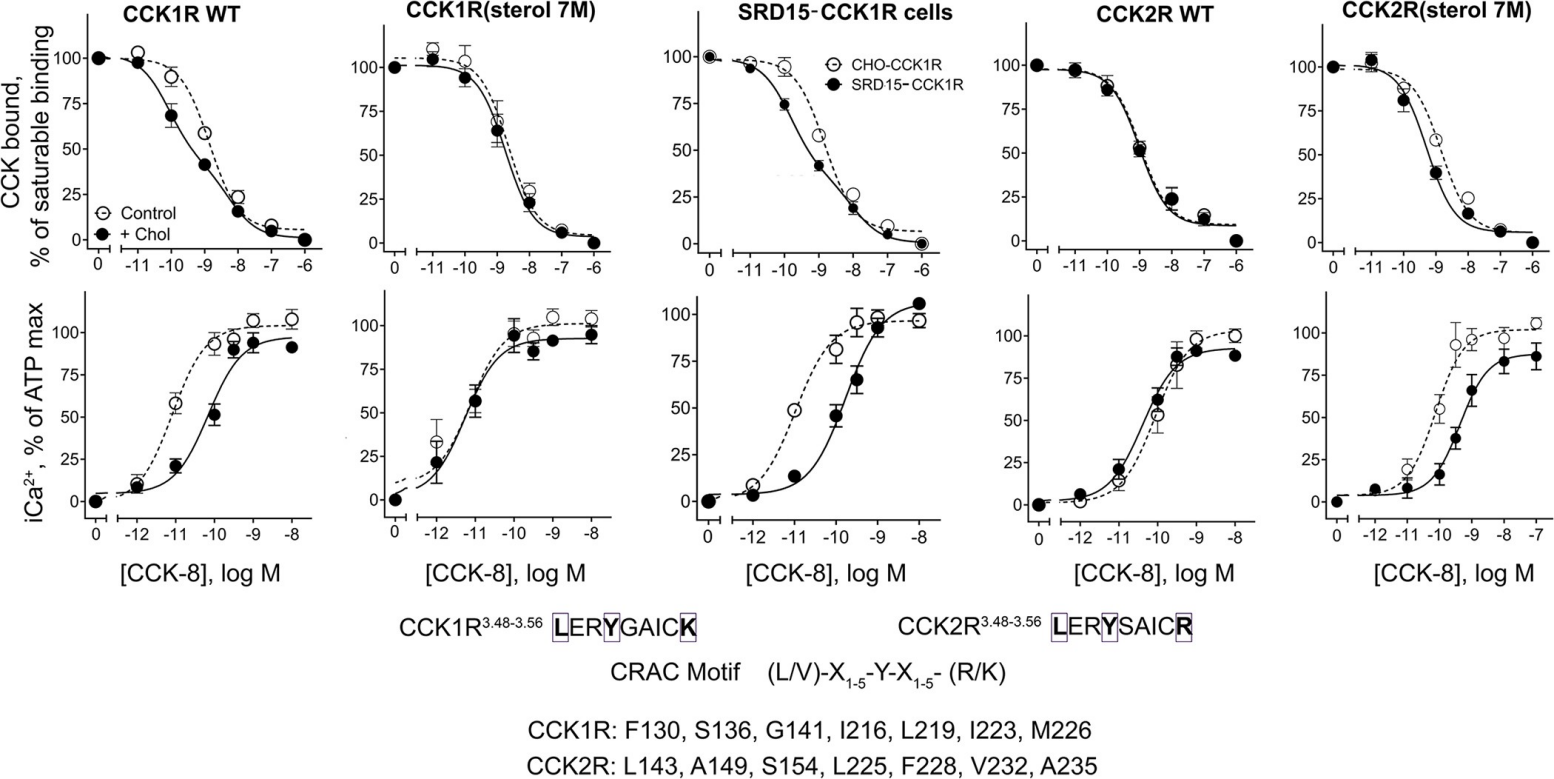
Characterization of CCK binding and biological activity at CRAC motif mutant constructs. Shown are competition-binding curves (top row) and intracellular calcium responses (bottom row) of CCK1R mutant constructs expressed in HEK293 cells in the absence and presence of enhanced membrane cholesterol. CCK binding and biological activity characteristics are also shown in SRD15 cells (genetically modified CHO cells with elevated levels of membrane cholesterol) engineered to express WT CCK1R (SRD15-CCK1R cells [10]). Binding values are reflected as percentages of saturable binding of CCK radioligand. Intracellular calcium responses are expressed as percentages of maximal responses to ATP (0.1 mM). The plots represent mean ± SEM of a minimum of 4 independent experiments performed in duplicate. Underlying data can be found in S1 Data.

Both CCK1R and CCK2R have consensus CRAC motifs in analogous positions low in TM3, yet CCK2R is not sensitive to increased cholesterol [10,11] (Fig 2). There are 7 surface residues predicted to be within 8 Å of Y140 that are different in CCK1R and CCK2R, (CCK1R/CCK2R: F130/L143, S136/A149, G141/S154, I216/L225, L219/F228, I223/V232, and M226/A235). Those residues were exchanged between CCK1R and CCK2R. The resulting receptor constructs, CCK1R(sterol 7M) and CCK2R(sterol 7M) (Fig 2) were characterized for affinity, calcium mobilization response, and cholesterol sensitivity following matched expression in HEK293 cells (Fig 2 and Table 1). Remarkably, exchange of these motifs reversed the functional sensitivity of these receptor constructs to increased cholesterol when stimulated with CCK-8. Unlike CCK1R(Y140A), which was cholesterol insensitive, but exhibited increased binding affinity and reduced calcium responsiveness relative to WT CCK1R in a normal membrane environment (Fig 1 and Table 1), the CCK1R(sterol 7M) construct was cholesterol insensitive, but displayed CCK-8 binding affinity (Fig 2, top row) and CCK-8-stimulated intracellular calcium responses (Fig 2, bottom row) equivalent to WT CCK1R in a normal membrane environment. In contrast, the CCK2R(sterol 7M) construct gained cholesterol sensitivity, displaying increased CCK-8 affinity but reduced potency in intracellular calcium mobilization. The functional characteristics observed for WT CCK1R in the HEK293 cell line when cholesterol was enriched were also seen in the CCK1R-expressing SRD15 mutant CHO cell line that had been genetically engineered to express high levels of cholesterol [10] (Fig 2 and Table 1).

### Structures of CCK1R(Y140A) and CCK1R(sterol 7M)

**Fig 3:**
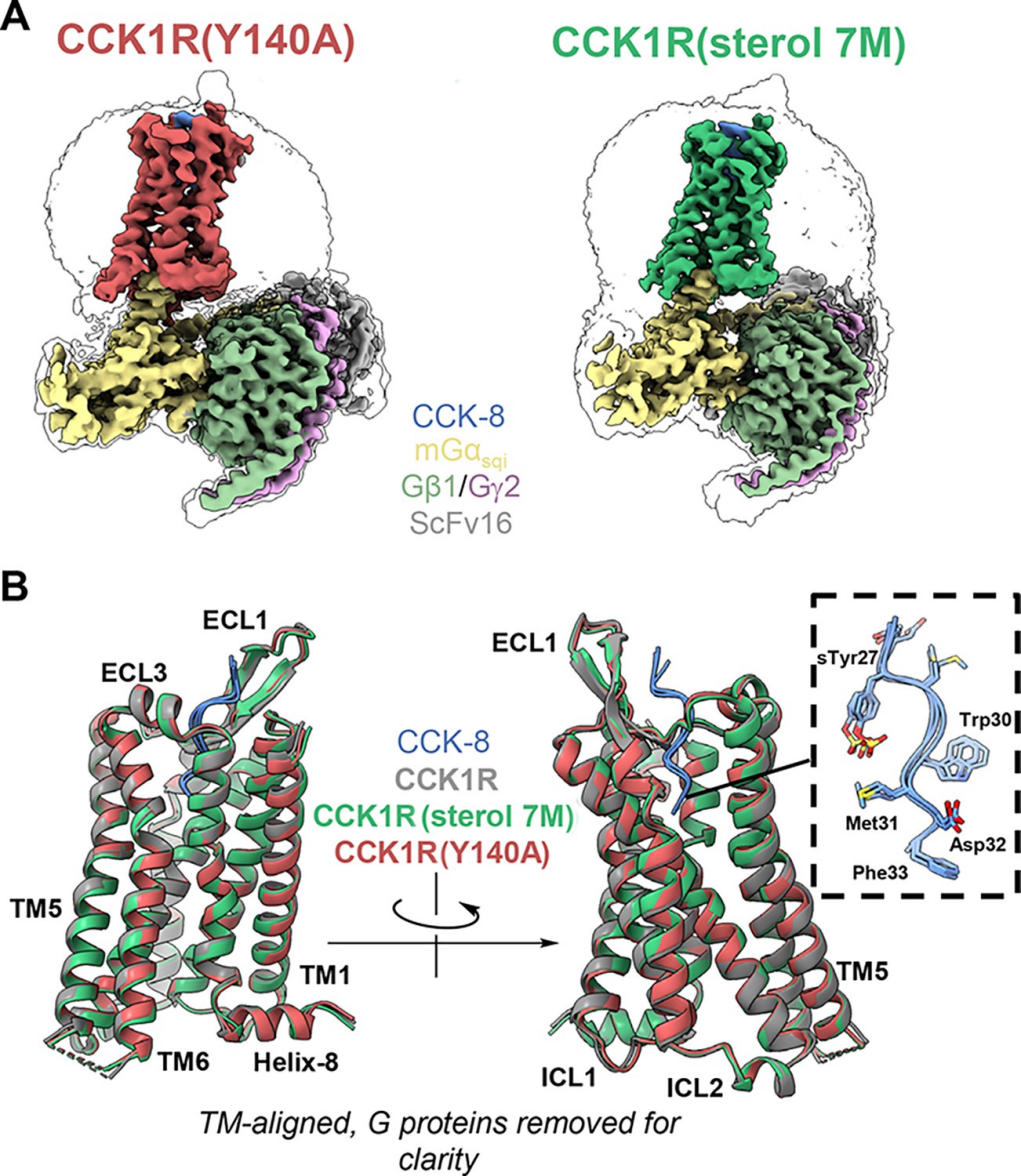
CryoEM structures of CCK1R(Y140A) and CCK1R(sterol 7M). (A) Shown are cryo-EM structures of the complexes containing CCK-8 and cholesterol-insensitive CCK1R constructs, CCK1R(Y140A) and CCK1R(sterol 7M), with Gq mimic. The colored surfaces show the maps at high threshold, and the transparent silhouettes show the maps at low threshold, allowing for visualization of the micelles. (B) Models of wild-type CCK1R (colored gray), CCK1R(sterol 7M) (colored green), and CCK1R(Y140A) (colored red) aligned on the transmembrane domains. The inset figure shows the close alignment of CCK-8 peptide among the 3 models. Structures were not different from the analogous structure of WT CCK1R previously reported [12], other than the mutations noted. Resolution was 2.51 Å for the CCK1R(sterol 7M) complex and 2.59 Å for the CCK1R(Y140A) complex (see S4 Fig). Atomic coordinates and cryo-EM density maps for CCK-8 bound CCK1R(sterol 7M) and CCK1R(Y140A) have been deposited in the PDB under accession numbers 9BKK and 9BKJ and Electron Microscopy Data Bank entries EMD-44643 and EMD-44642, respectively.

Shown in Fig 3 are the consensus cryo-EM structures of complexes of the pharmacologically distinct cholesterol-insensitive CCK1R mutants, CCK1R(Y140A) and CCK1R(sterol 7M), in the presence of CCK-8 and Gq mimic. Resolution of these complexes was 2.51 Å for CCK1R(sterol 7M) and 2.59 Å for CCK1R(Y140A) (see S4 Fig). These structures required stabilization of the receptor complex through use of a guanine nucleotide-insensitive G protein analogue [22], with further stabilization of the bound heterotrimer by scFv16 that spans the αN helix of the chimeric Gα subunit and Gβ. Moreover, the complexes were solubilized with lauryl maltose neopentyl glycol supplemented with cholesteryl hemisuccinate [12]. Under these conditions, there were no substantial structural differences between these constructs and when compared with WT CCK1R [12] (approximately 0.7 Å Cα RMSD for receptor residues for both CCK1R(Y140A) and CCK1R(sterol 7M) compared to CCK1R), other than the presence of the specific mutant residues that could underlie the observed functional dissimilarities. This might indicate that the differences are primarily related to the rates of G protein turnover [23], events that are transient by their nature, and may be incompatible with the G protein stabilization required for elucidation of static structures using standard cryo-EM techniques for membrane proteins. As such, we next investigated the possible dynamic events at the extracellular surface involved in natural ligand binding and at the cytosolic surface involved in G protein association that might not have been captured in this type of stable structure.

### Cholesterol and CCK1R mutants alter kinetics and pose of CCK binding

**Fig 4:**
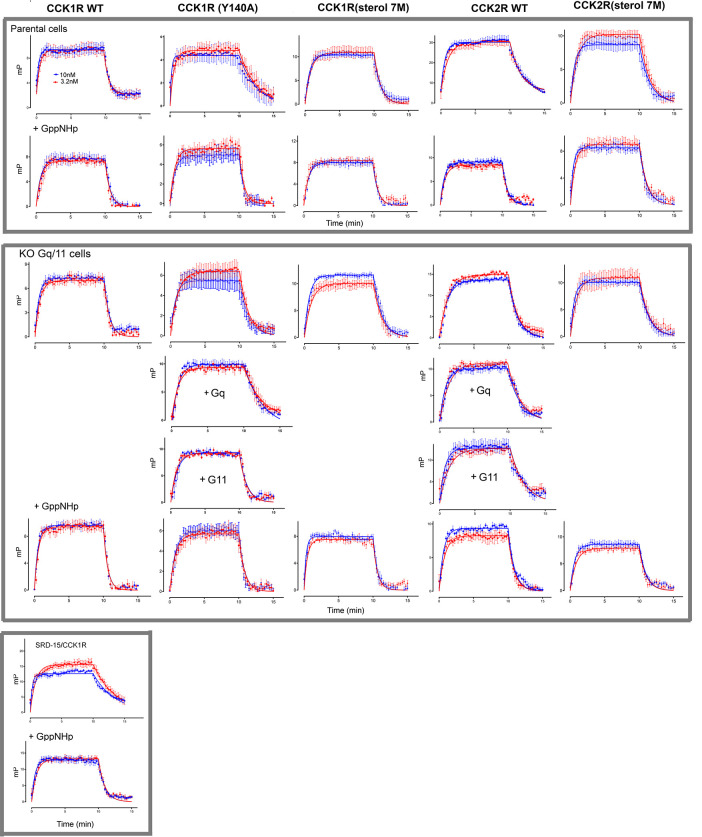
Kinetic binding profiles of alexa^488^-Gly-[(Nle^28,31^)CCK-26-33] in CCK receptor constructs. Top 2 rows show the profiles of CCK association and dissociation to receptor constructs expressed in control parental cells in the absence and presence of GppNHp. Middle 2 rows show analogous profiles in the CCK receptor constructs expressed in Gq KO cells. The bottom 2 rows show analogous data for the KO cells in which Gq or G11 are reintroduced, as well as the SRD15-CCK1R cell line [10]. Values are expressed as mean ± SEM from a minimum of 4 experiments performed in triplicate. Underlying data can be found in S1 Data.

Fluorescence polarization (FP) [8] was used to monitor the kinetics of association and dissociation of the CCK-like fluorescent probe, alexa^488^-Gly-[(Nle^28,31^)CCK-26-33] [24], at individual receptor constructs, and in CCK1R-expressing cells with elevated cholesterol (Fig 4 and Table 2). The rates of CCK dissociation (off rates) were significantly slower at CCK1R in the setting of elevated cholesterol (SRD15 cells expressing CCK1R [10]) and in the construct mimicking the high cholesterol state, CCK1R(Y140A), than at WT CCK1R, and this was correlated with a higher calculated affinity. Similarly, the CCK off rate was slower at WT CCK2R relative to WT CCK1R. Interestingly, the CCK1R(sterol 7M) construct exhibited an off rate for CCK that was intermediate between that of CCK1R and CCK1R(Y140A), but also with a trend toward a slower on rate (Kon) and a calculated affinity similar to CCK1R, while the cholesterol-sensitive CCK2R(sterol 7M) construct displayed significantly faster ligand dissociation than CCK2R.

**Table 2:** Kinetic constants of alexa^488^-Gly-[(Nle^28,31^)CCK-26-33] binding to CCKR constructs.

	-GppNHp				+GppNHp				*P values compared to-GppNHp K_on_/K_off_/pK_i_
	K_on_ X10^8^ M^-1^ min^-1^	K_off_ min^-1^	pK_i_	n	K_on_	K_off_	pK_i_	n	
***Parental cells***									
CCK1R WT	2.4±0.5	1.5±0.1	8.1±0.1	8	0.5±0.2*	1.8±0.2	7.5±0.1*	5	0.011/0.354/0.006
CCK1R(Y140A) *P* values *vs* CCK1R	4.9±1.0 0.083	0.5±0.1# 0.001	9.0±0.1# 0.001	8	0.5±0.1*	1.6±0.1*	7.5±0.1*	5	0.001/0.003/0.002
CCK1R(sterol 7M) *P* values *vs* CCK1R	1.0±0.4 0.059	1.0±0.2# 0.029	7.9±0.2 0.282	6	0.5±0.1	1.4±0.1	7.6±0.1	5	0.537/0.177/0.329
CCK2R WT *P* values *vs* CCK1R	3.0±1.0 0.755	0.4±0.04# 0.001	8.8±0.2# 0.008	6	0.4±0.1*	1.0±0.1*	7.6±0.1*	5	0.004/0.004/0.004
CCK2R(sterol 7M) *P* values *vs* CCK2R	3.5±1.9 0.699	0.6±0.1# 0.026	8.3±0.3 0.288	6	1.8±0.6	1.0±0.2	8.2±0.2	5	0.876/0.149/0.530
***KO Gq/11 cells***									
CCK1R WT *P* values *vs* parental	1.3±0.6 0.189	1.4±0.3 0.072	7.7±0.2 0.152	7	0.5±0.1	1.6±0.2	7.5±0.2	5	0.530/0.202/0.530
CCK1R(Y140A) *P* values *vs* parental	0.9±0.3^@^ 0.001	0.9±0.1^@^ 0.021	7.9±0.1^@^ 0.0002	8	3.1±2.0	1.2±0.2	8.0±0.3	5	0.742/0.093/0.622
CCK1R(Y140A) KO Gq/11 + Gq *P* values *vs* CCK1R(Y140A)KO Gq/11	1.9±0.4$ 0.028	0.3±0.03$ 0.004	8.7±0.2$ 0.016	4					
CCK1R(Y140A) KO Gq/11 + G11 *P* values *vs* CCK1R(Y140A) KO Gq/11	1.7±0.5 0.461	0.8±0.03 0.683	8.2±0.2 0.283	4					
CCK1R(sterol 7M) *P* values *vs* parental	0.7±0.2 >0.999	0.8±0.1 0.537	7.9±0.2 0.931	6	1.8±0.2	1.7±0.2*	7.9±0.2	5	0.309/0.008/0.841
CCK2R WT *P* values *vs* parental	0.9±0.5 0.052	0.8±0.1@ 0.004	7.7±0.3@ 0.015	5	0.4±0.1	1.1±0.1	7.6±0.1	5	0.691/0.151/0.841
CCK2R KO Gq/11 + Gq *P* values *vs* CCK2R KO Gq/11	0.8±0.3 0.413	0.5±0.04$ 0.032	8.1±0.2 0.191	4					
CCK2R KO Gq/11 + G11 *P* values *vs* CCK2R KO Gq/11	1.1±0.6 0.556	0.5±0.1$ 0.032	8.1±0.3 0.286	4					
CCK2R(sterol 7M) *P* values *vs* parental	2.2±1.2 0.628	0.7±0.04 0.181	8.1±0.3 0.534	6	0.6±0.2	1.0±0.1*	7.7±0.1	5	0.662/0.009/0.662
***SRD15-CCK1R cells***									
SRD15-CCK1R cells *P* values *vs* CCK1R	1.7±0.4 0.463	0.5±0.1^#^ 0.001	8.5±0.1# 0.003	7	1.9±1.1	1.0±0.1*	7.9±0.2	5	0.429/0.004/0.178

Values are expressed as mean ± SEM from “n” observations performed in triplicate. Data were analyzed with unpaired *t* test with Mann–Whitney post-test.

* *p* < 0.05, significantly different from -GppNHp for cell line.

^#^ *p* < 0.05, significantly different from CCK1R-bearing cell line.

@ *p* < 0.05, significantly different from same construct in parental cells.

$ *p* < 0.05, significantly different from noted control with G protein KO.

The agonist ligand off rate of many receptors is correlated with G protein engagement [25,26], with slow off rates and longer ligand occupancy times linked to reduced G protein activation (i.e., slower G protein turnover and decreased signaling) [23]. Decoupling of G proteins via addition of the non-hydrolyzable nucleotide GppNHp (10 μM) slowed the Kon for all receptor constructs (Fig 4, top panel and Table 2), suggesting that G protein interaction might promote increased conformational dynamics of the extracellular domain of the receptor to facilitate initial peptide binding. In contrast, G protein uncoupling led to increased alexa^488^-Gly-[(Nle^28,31^)CCK-26-33] dissociation from all receptor constructs, except for the WT CCK1R in a normal membrane environment, such that the CCK1R mutant receptors displayed equivalent kinetics to WT CCK1R (Fig 4, top panel). Of note, G protein uncoupling also resulted in a more rapid ligand off rate from WT CCK1R in the elevated membrane cholesterol environment present in the SRD15 cells [10]. Both the CCK2R and CCK2R(sterol 7M) exhibited similar binding kinetics with excess GppNHp, albeit that the off rate for the CCK2R constructs remained slower than was observed for the CCK1R constructs. Collectively, these data suggest that the impact of cholesterol on ligand binding kinetics is primarily G protein dependent.

In previous work, we revealed that, in contrast to the reduced potency of CCK-mediated calcium mobilization, increased cholesterol enhanced peptide potency for Gs-mediated cAMP production [12]. As such, we probed the extent to which kinetic differences between receptor constructs were dependent upon Gq/11 proteins through use of HEK 293 cells genetically engineered to lack their expression [27]. With the exception of the CCK1R(sterol 7M), absence of Gq/11 proteins was associated with a trend toward slower Kon values (Fig 4, middle panel and Table 2) that was significant for the CCK1R(Y140A) mutant. There was a parallel increase in the off rate for CCK1R(Y140A) and CCK2R, and a decreased calculated affinity, suggesting that the slow off rate for these receptors is largely dependent upon presence of Gq/11 proteins. In contrast, the alexa^488^-Gly-[(Nle^28,31^)CCK-26-33] kinetics of the cholesterol-insensitive CCK1R(sterol 7M) construct were not altered by absence of Gq/11 proteins. There were no significant differences in the peptide kinetics between cells with and without Gq/11 proteins in the presence of excess GppNHp, albeit that large errors in the Kon estimates determined in the KO cells made interpretation of potential effects on this parameter problematic. To further probe the importance of Gq or G11 proteins, we performed additional experiments on the CCK1R(Y140A) mutant and CCK2R, the 2 constructs that were most impacted by Gq/11 deletion, where either Gq or G11 was transfected into the KO cells (Fig 4 and Table 2). At the CCK2R, reintroduction of either Gq or G11 reversed the increased ligand dissociation rate observed in the KO cells. In contrast, transfection of Gq, but not G11, reversed the higher off rate at CCK1R(Y140A), suggesting that there may be subtle differences in the roles of the 2 closely related Gα protein subtypes at the CCK1R.

One possible determinant of the stability and duration of peptide occupation is the nature of the peptide engagement with the receptor. Indeed, distinct poses of CCK when docked to CCK1R have been proposed in the literature based on mutagenesis and photoaffinity crosslinking [8,28–31], and there are robust data to support the hypothesis that these poses could be affected by cholesterol [7]. At the CCK1R, photoaffinity cross-linking of a CCK-8 analogue with a photoactive residue at the C terminus (residue 33 of CCK(1–33)) indicated that this residue has a predominantly superficial orientation sitting at the surface of the lipid bilayer adjacent to TM1 [28,31], whereas mutagenesis data also support a deeper pose, similar to that proposed for CCK2R [29,30]. The recent cryo-EM structures of CCK-8 bound to either CCK1R or CCK2R demonstrated an equivalent “deep” pose at both receptors for the CCK-8 C terminus when in stable complex with G protein in the nucleotide free (G_0_) state [25,26], as also seen for the CCK1R(Y140A) and CCK1R(sterol 7M) constructs described above.

These data are consistent with a model where the binding of CCK-8 is dynamic, exchanging between shallow and deep poses, particularly at the CCK1R, but where the deep pose can be stabilized by G protein interaction. In this model, cholesterol may act by slowing the off rate of Gq/11 proteins, which in turn can allosterically slow peptide dissociation. At the WT CCK1R, the peptide C terminus is likely to form more transient interactions in the deeper pose under conditions where Gq/11 proteins undergo rapid nucleotide exchange to become activated and dissociated from the receptor. In structural studies, agonists in active receptor complexes are trapped in the high affinity, nucleotide free, G protein-bound state where the peptide is stably engaged in the deep pose. To test this hypothesis more explicitly, we performed photoaffinity crosslinking and biophysical studies of solvent exposure of the C-terminal residue of analogues of CCK-8 with key receptor constructs.

**Fig 5:**
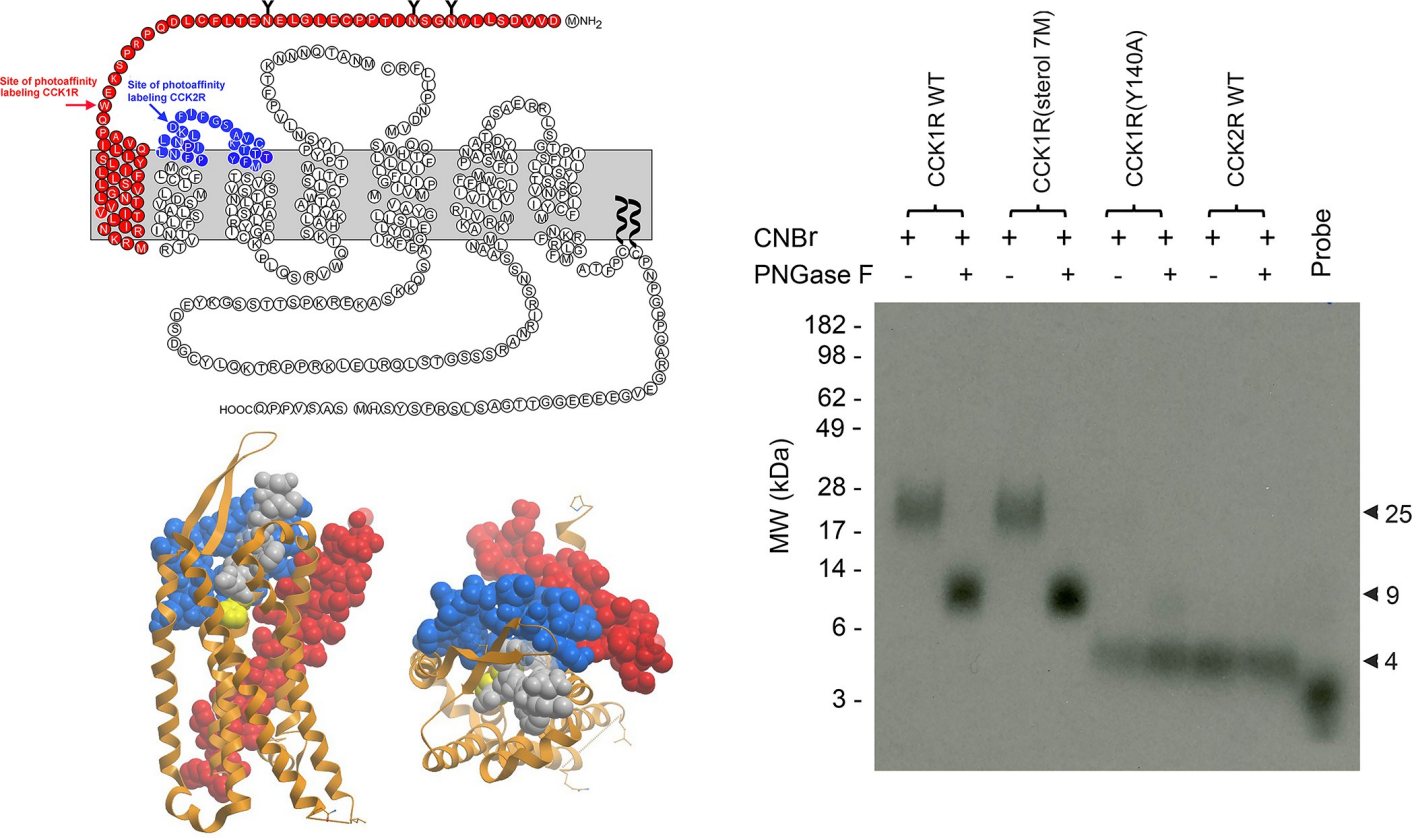
Photoaffinity labeling of CCK receptor constructs. Shown is a representative autoradiograph of a 10% NuPAGE gel used to separate the products of CNBr cleavage of CCK receptor constructs photoaffinity labeled through their C-terminal benzoyl-phenylalanine moiety, as well as the migration of probe alone. Alternate lanes show the products deglycosylated with PNGase F, establishing the fragments labeled in WT CCK1R and CCK1R(sterol 7M) as glycosylated, whereas those labeled in WT CCK2R and CCK1R(Y140A) as a much smaller and non-glycosylated. Also shown are the CNBr fragments of CCK1R in a 2D snake plot, as well as these fragments highlighted in the cryo-EM structure of CCK1R with views form the side and top [12]. The fragment highlighted in red is that labeled in CCK1R [31] and the fragment highlighted in blue is analogous to that labeled in CCK2R [28]. Underlying data can be found in S1 Data. Uncropped gel autoradiograph image can be found in S1 Raw Images.

Intrinsic photoaffinity labeling is a technique in which proximity between distinct residues within a peptide hormone pharmacophore and its receptor can be directly established. This has been applied to the residue at the C terminus of CCK-8, via substitution of photolabile residues for the native Phe^33^, as docked at CCK1R [28,31] and CCK2R [28]. Using 2 different photochemical groups, nitro-phenylalanine [31] and benzoyl-phenylalanine [28], the site of covalent labeling of CCK1R was Trp39 [31] and of CCK2R was Thr119 [28], with the latter consistent with the deep pose present in the cryo-EM structures of both CCK-bound CCK1R and CCK2R as noted above [12,13,32] (Fig 5). In contrast, the C-terminal residue of CCK-8 in the active complex structures is spatially distant from the CNBr segment containing the top of TM1 that is covalently labeled in CCK1R expressed in cells with a normal (lower cholesterol) membrane environment (Fig 5). We hypothesized that, under these conditions, the CCK peptide at CCK1R is more dynamic such that the more superficial pose is favored in the cross-linking studies.

To further explore this, we applied photoaffinity labeling with (^125^I-des-amino-Tyr)-Gly-[(Nle^28,31^)CCK(26–32)]-Bpa^33^ to the cholesterol-insensitive CCK1R constructs described above, CCK1R(Y140A) mimicking the high cholesterol state and CCK1R(sterol 7M) mimicking the basal state, which revealed differential covalent labeling (Fig 5). The pattern from photoaffinity labeling of the CCK1R(sterol 7M) construct was the same as previously demonstrated for WT CCK1R in which Bpa^33^ covalently labeled Trp39, a residue just above TM1 in the large glycosylated CNBr fragment with mass of 7952 plus that of the carbohydrate (Fig 5) [28,31]. In contrast, the CNBr fragment pattern from photoaffinity labeling of the CCK1R(Y140A) construct was clearly distinct, labeling of a much smaller and non-glycosylated fragment of the receptor. The best candidate corresponding with this labeled band has mass 2961 that is analogous to the CNBr fragment of CCK2R that was previously labeled with this probe on Thr119 [28] (Fig 5). The cross-linking patterns are consistent with the slow dissociation rates observed for the CCK1R(Y140A) and CCK2R being associated with a more stable engagement with the deeper pose.

**Fig 6:**
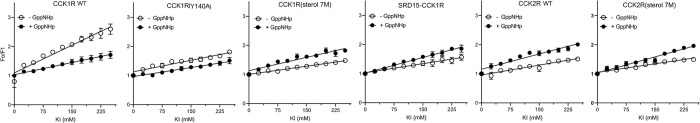
Fluorescence quenching of Gly-[(Nle^28,31^)CCK-26-32]-aladan^33^ bound to CCK receptor constructs. Shown are the plots of collisional quenching of aladan^33^-containing probe bound to the noted CCK receptor constructs using KI in the absence or presence of GppNHp. Values are expressed as mean ± SEM from 3 independent experiments. Underlying data can be found in S1 Data.

As a further measure of potential differences in peptide dynamics, we probed the local microenvironment of the C terminus of CCK-8 with different receptor constructs using an analogue substituted with dansylalanine (aladan) into the position of Phe^33^ [10,33,34]. This probe was also fully biologically active and bound to CCK1R with normal affinity [10,33,34]. We performed potassium iodide (KI) quenching experiments similar to those we previously reported for WT CCK receptors [10,33,34]. These data report on aqueous accessibility of the probe and results are shown in Fig 6 and Table 3. All receptor constructs displayed similar, low levels of KI quenching in the G protein-uncoupled state induced by excess GppNHp. However, in native cell membranes without nucleotide, only the WT CCK1R displayed high levels of quenching, consistent with a more prominent, superficial pose and greater peptide dynamics providing greater aqueous accessibility. Quenching studies on both the high cholesterol mimetic mutant (Y140A) and on CCK1R expressed in the high cholesterol-containing SRD15 cells [10] revealed low levels of quenching, consistent with a stable, more buried pose that was also similar to the results with the WT CCK2R. Surprisingly, the KI quenching of CCK1R(sterol 7M) was like that at CCK2R, rather than being similar to CCK1R, and this was also true for the CCK2R(sterol 7M). However, it is noteworthy that the dissociation kinetics of the alexa^488^-Gly-[(Nle^28,31^)CCK-26-33] peptide at both the sterol 7M chimeras were still slower than the fast dissociation observed with the WT CCK1R, potentially accounting for the differences in the KI quenching experiments.

**Table 3:** Fluorescence quenching constants of Gly-[(Nle^28,31^)CCK-26-32]-aladan^33^ probe at CCK receptor constructs expressed in HEK-293 cell lines or CHO-K1 cells (SRD15-CCK1R cells).

Construct	-GppNHp (active state)	+GppNHp (uncoupled state)	Comparison of active vs. uncoupled states n, *P* values	Comparison with CCK1R (active state) *P* values
CCK1R WT	6.3±0.6	2.8±0.5*	5, 0.008	
CCK1R(Y140A)	2.6±0.1^#^	2.1±0.3	3, 0.400	0.0002
CCK1R(sterol 7M)	1.9±0.1^#^	3.1±0.4	3, 0.100	<0.0001
SRD15-CCK1R	2.1±0.4^#^	3.6±0.4	4, 0.057	<0.0001
CCK2R WT	2.3±0.2^#^	3.5±0.1*	4, 0.029	<0.0001
CCK2R(sterol 7M)	2.0±0.03	3.7±0.5	3, 0.100	0.996

Values are expressed as mean ± SEM from “n” observations performed. Data were analyzed with one-way ANOVA with Tukey’s multiple comparison test.

* *p* < 0.05, significantly different from active state of the same construct.

^#^ *p* < 0.05, significantly different from CCK1R WT. Underlying data are found in S1 Data.

### Impact of membrane cholesterol on events in the G protein cycle

As discussed above, the data with the cholesterol-insensitive CCK1R mutants and CCK1R in high cholesterol are consistent with a primary effect of cholesterol on peptide kinetics that is mediated by changes in G protein interactions. More specifically, the data suggested that cholesterol might slow the activation and turnover of Gq/11 proteins leading to both slow CCK-8 dissociation and to reduced Gq/11-dependent signaling. If this were indeed the case, we reasoned that there should be parallel changes in the rates of agonist-mediated conformational change and G protein activation. This hypothesis was empirically assessed using Gq conformational sensors, and the TRUPATH assay for Gq or G11 dissociation (as a surrogate for activation) [35].

**Fig 7:**
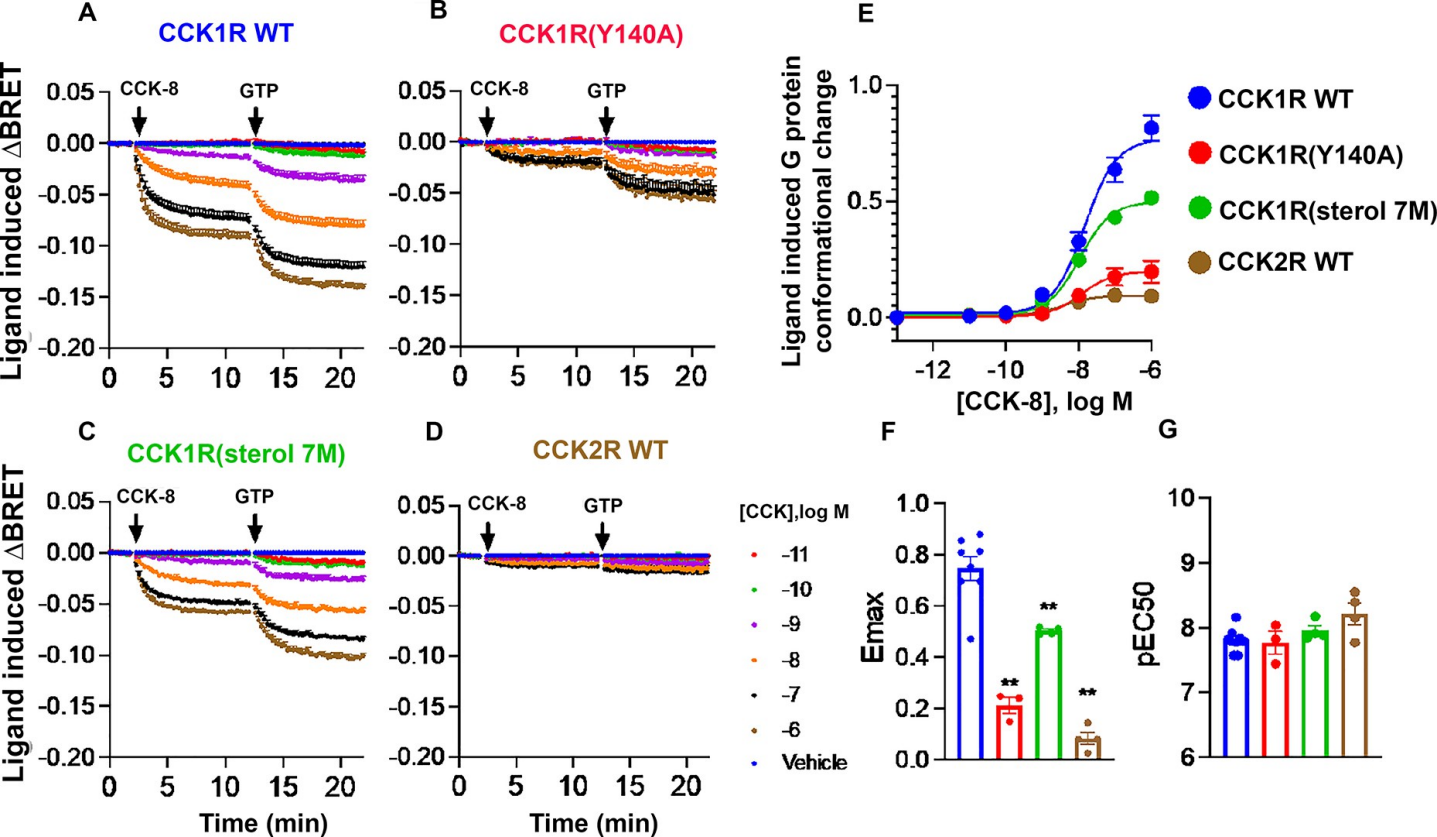
Membrane BRET studies to evaluate G protein conformational changes. Shown in the top row are the kinetic traces of CCK ligand-induced changes in the BRET signal collected between Gαq-Nlu and Gγ-Venus transiently transfected in Gq/11 KO cells expressing CCK receptor constructs. Shown in separate graphs are summaries of the degrees of conformational change induced by CCK and GTP, and their concentration dependency. Values are expressed as mean ± SEM from a minimum of 3 independent experiments. Data were analyzed by one-way ANOVA with Dunnett’s multiple comparison test. * *p* < 0.05; ** *p* < 0.01. Underlying data can be found in S1 Data.

In the initial studies, we used membrane-based Gq protein conformation assays that are sensitive to the distance between Gα and Gγ to determine whether CCK1R and CCK2R can promote different G protein conformations upon activation. This membrane-based system allowed us to control nucleotide concentration, thus stabilizing the high affinity tertiary complex. Basal signals were similar for all the cell lines, and binding of CCK-8 produced a Gq conformational change that resulted in a decrease in BRET signal at the different CCKR constructs. However, the population-based peptide-induced change in BRET signal was markedly smaller at CCK2R compared to CCK1R, consistent with differential engagement with Gq proteins at these receptors (Fig 7A, 7D and 7E). Notably, the high cholesterol state mimic CCK1R(Y140A) exhibited significantly reduced magnitude of G protein conformational change compared to WT CCK1R, and showed a more “CCK2R-like” phenotype (Fig 7B and 7E). Of interest, the CCK1R(sterol 7M) construct also exhibited a smaller CCK-8-induced G protein conformational change signal compared to CCK1R, again consistent with this construct exhibiting an intermediate phenotype between WT CCK1R and high cholesterol mimetic states, despite a predominant lower cholesterol WT CCK1R phenotype in equilibrium binding and intracellular calcium assays (Fig 7C and 7E).

**Fig 8:**
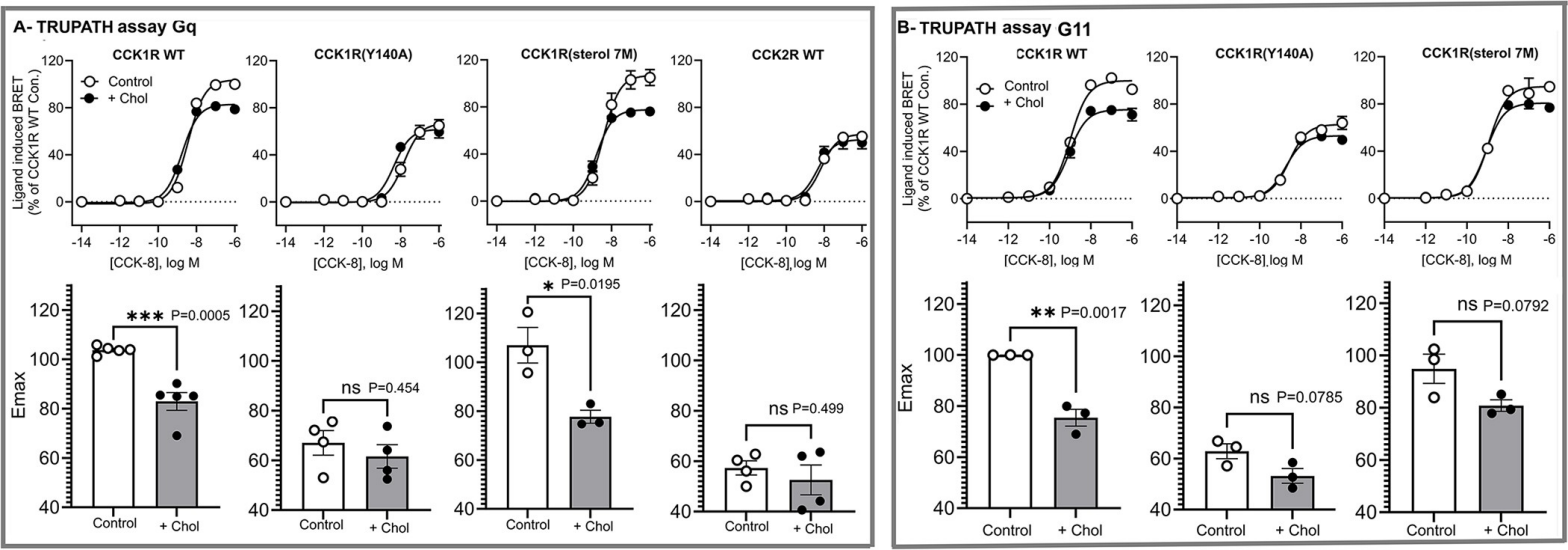
TRUPATH assays of G protein activation at CCK receptor constructs. Shown are the CCK ligand concentration-dependent changes in signal obtained between Gαq-Rluc8, Gβ3, and Gɤ9-GFP2 in Gq/11 KO cells expressing CCK receptor constructs (panel A); and between Gα11-Rluc8, Gβ3, and Gɤ9-GFP2 in Gq/11 KO cells expressing CCK receptor constructs (panel B). Cells were studied in the absence and presence of enriched cholesterol. Bar graphs reflect E_max_ values for control and elevated cholesterol conditions. Values are expressed as mean ± SEM from a minimum of 3 independent experiments performed in triplicate. Data were analyzed by one-way ANOVA with Tukey’s multiple comparison test. * *p* < 0.05;** *p* < 0.01; *** *p* < 0.001. Underlying data can be found in S1 Data.

Next, we evaluated steady-state G protein turnover using the intact cell TRUPATH G protein dissociation assay (Fig 8). Cholesterol enrichment reduced the E_max_ of the Gq-TRUPATH profile on WT CCK1R, indicating a decrease in Gq protein dissociation/activation. Consistent with the limited population level change in Gq conformation (Fig 8A), there was a much smaller CCK-8-mediated Gq dissociation at the CCK2R compared to that at CCK1R, and it was unaffected by increased cholesterol (Fig 8A). Similarly, the high cholesterol mimetic CCK1R(Y140A) construct exhibited lower CCK-8-mediated Gq dissociation compared to WT receptor, with this profile unchanged by cholesterol enhancement. Intriguingly, while the maximal peptide-induced Gq dissociation of the CCK1R(sterol 7M) was similar to the WT CCK1R, cholesterol enrichment reduced the E_max_ of Gq coupling to this receptor construct, to a similar extent to that observed with the WT receptor (Fig 8A). This observation was inconsistent with the functional observation where CCK1R(sterol 7M)-mediated intracellular calcium mobilization was not altered by increased cholesterol levels.

Nonetheless, both Gq and G11 activation can trigger intracellular calcium signaling and therefore, we investigated CCK-8-mediated G11 dissociation using TRUPATH (Fig 8B). Under basal conditions, there was a similar level of G11 dissociation for the WT CCK1R and the CCK1R(sterol 7M), with reduced E_max_ at the Y140A mutant. However, while membrane cholesterol enrichment reduced E_max_ for G11 activation at WT CCK1R, there was limited effect at the CCK1R(sterol 7M) or CCK1R(Y140A) constructs. Consistent with the differential effects of reintroduction of Gq versus G11 in alexa^488^-Gly-[(Nle^28,31^)CCK-26-33] kinetics in Gq/11 knockout cells (Fig 4 and Table 2), these data suggest that there might be a distinct mode of engagement with Gq versus G11 at CCK1R.

Collectively, the data support a model of differential CCK-8 interaction with the CCK1R and CCK2R where the peptide is more dynamic at the CCK1R, transitioning between a superficial engagement pose and a deeper pose that is likely required for G protein activation, whereas CCK-8 can more readily engage with the deeper pocket at CCK2R. The more transient interaction of the peptide with the deeper pose likely leads to a faster rate of Gq/11 protein turnover and consequently a more potent downstream intracellular calcium response. CCK2R has a more stable engagement of the peptide in the deep pose with a slower off rate that is associated with a slower rate of Gq/11 protein activation and turnover, and lower potency for calcium mobilization. While the slow CCK-8 off rate is partially dependent upon G protein, this is slower than the rate of peptide dissociation at the CCK1R even when the receptors are decoupled from G proteins. The data are also consistent with the possibility of CCK2R having a stronger interaction with cholesterol within the conserved CRAC motif in TM3 that is sensitive to changes in cholesterol concentration at the CCK1R. Increased cholesterol at the CCK1R appears to be primarily associated with stabilization of the interaction of Gq/11 proteins with the receptor reducing G protein dissociation/activation and reducing potency in downstream signaling. This, in turn, reduces the dynamics of the CCK-8 peptide and stabilizes the deeper pose of the C terminus of the peptide within the TM core, that is reflected in the reduced off rate. The CCK1R(Y140A) mutant mimics the high cholesterol state by a similar mechanism that is dependent upon slower Gq/11 turnover. The sterol 7M constructs appear to differ from WT receptors in the ability of cholesterol to alter the interaction with Gq/11 proteins, and this is particularly evident through the lack of altered peptide kinetics in cells genetically depleted of Gq/11 proteins, although this may be partly dependent upon the relative expression of Gq versus G11 proteins. While counter intuitive to the slower release of Gq/11 proteins, the increased stability of CCK-8 in the deeper pose with high cholesterol likely also further increases the recruitment and turnover of the non-canonical Gs protein that is reflected in the observed higher potency for cAMP production [12]. Moreover, as the deep pose is linked to stable G protein engagement, it is therefore not surprising that only this pose is present in structures of active CCK1R and CCK2R that are engineered to stabilize the G protein-bound complex in the high-affinity, G_0_ (nucleotide-free)-bound state. This CRAC motif is proximal to intracellular loop 2 (ICL2) of the receptor, which is a key GPCR domain for G protein interaction and activation [36–38], providing a potential mechanistic basis for how cholesterol might alter G protein engagement via modifying the dynamics of ICL2.

A corollary to the above is that CCK peptides have a lower energy barrier to achieving the deep peptide binding pose required for Gq/11 engagement at the CCK2R relative to CCK1R, and this is consistent with the greater potency of non-sulphated CCK-8-DS and the C-terminal tetrapeptide, CCK-4, at inducing calcium mobilization at the CCK2R, with these peptides having substantially lower potency at CCK1R. Consequently, we wished to explore the impact of high cholesterol on binding affinity and potency in intracellular calcium mobilization of the non-sulfated CCK-8-DS and shorter CCK-4 peptides at the CCK1R.

### Impact of membrane cholesterol on non-sulphated CCK peptide binding to CCK1R

**Fig 9:**
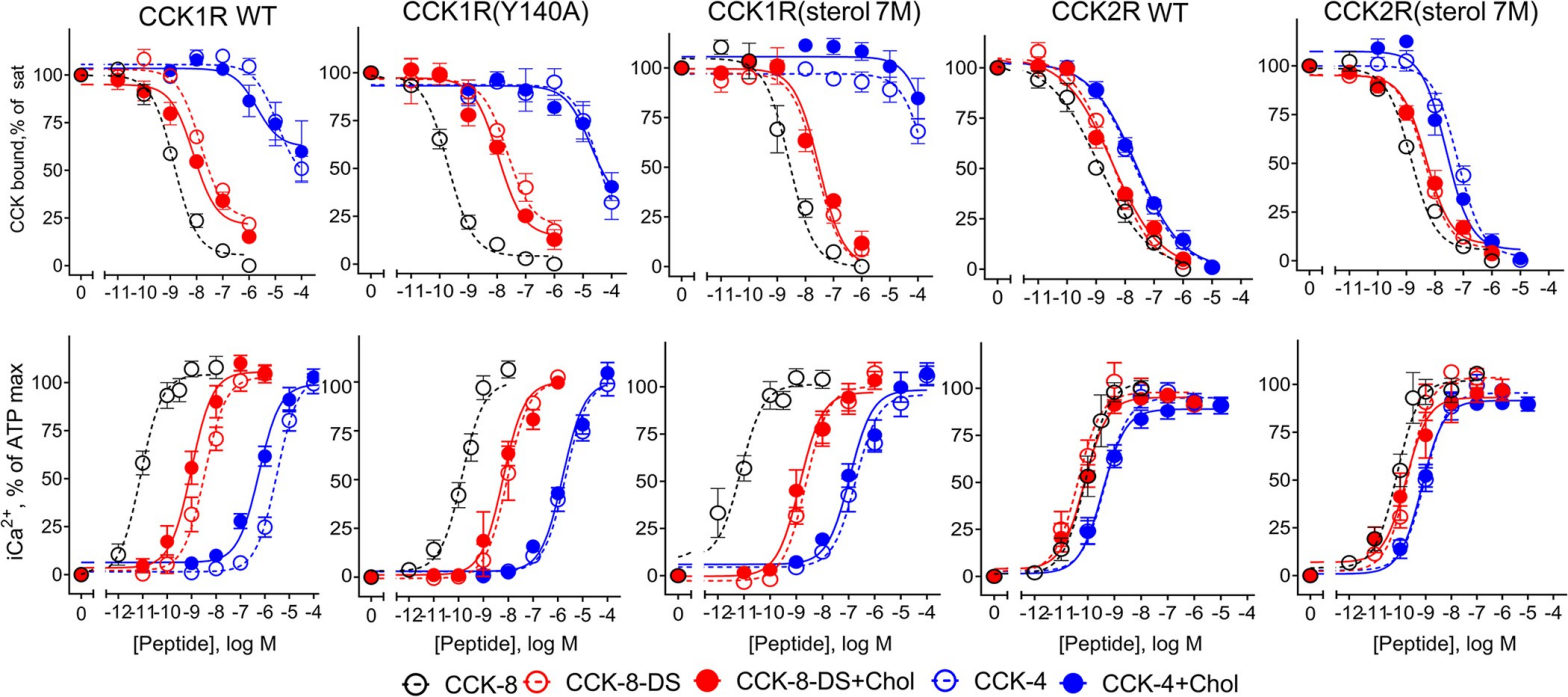
Structure-activity relationships of CCK peptide binding and biological activity. Shown are competition-binding curves (top row) and intracellular calcium responses (bottom row) of CCK variants expressed in HEK293 cells in the absence and presence of enhanced cholesterol. Binding values are reflected as percentages of saturable binding of CCK radioligand. Intracellular calcium responses are expressed as percentages of maximal responses to ATP (0.1 mM). The plots represent mean ± SEM of a minimum of 4 independent experiments performed in duplicate. Underlying data can be found in S1 Data.

As discussed above, the binding affinity and Gq/11-dependent functional potency of the non-sulfated CCK-8-DS and shorter CCK-4 are significantly lower than that of CCK-8 at CCK1R, while being similar to that of CCK-8 at CCK2R, as shown in Fig 9 and Table 4. Interestingly, while effects on binding affinity were minimal, increased cholesterol in cells expressing the WT CCK1R led to significant increases in peptide potency for both the CCK-8-DS and CCK-4 peptides. This was also true for the SRD15-CCK1R cell line, with depletion of cholesterol with MβCD reversing this effect (Table 4). These data indicate that the high cholesterol enhances the potency of non-sulfated CCK peptides at WT CCK1R, possibly by facilitating their engagement with the deep pocket of this receptor. Intriguingly, while responses to the non-sulfated peptides at the CCK1R(Y140A) and CCK1R(sterol 7M) remained cholesterol insensitive, peptide potency, particularly for CCK-4, was higher at the CCK1R(sterol 7M) mutant compared with CCK1R(Y140A). This suggests that the CCK1R(sterol 7M) mutant can support both increased recruitment and activation of Gq/11 proteins, while the CCK1R(Y140A) mutant is unable to fully mimic the impact of high cholesterol for these peptides.

**Table 4:** Binding affinity and biological activity of CCK analogues at CCK receptor constructs expressed in natural and cholesterol-enriched HEK-293 cells or CHO-K1 cells (SRD15-CCK1R cells).

Constructs	pIC_50_ (“n”)	*P* values compared to control	B_max_x10^5^sites/cell (“n”)	*P* values compared to control	iCa^2+^, pEC_50_ (“n”)	*P* values compared to control
***CCK-8-DS***						
CCK1R WT Control + Chol	7.3±0.1 (4) 7.5±0.2 (4)	0.685	0.6±0.2 (4) 0.8±0.2 (4)	0.485	8.5±0.2 (6) 9.0±0.2 (6) *	0.041
CCK1R(sterol 7M) Control + Chol	7.6±0.1 (4) 7.8±0.1 (4)	0.685	0.2±0.1 (4) 0.3±0.1 (4)	0.343	8.6±0.2 (4) 8.8±0.3 (4)	0.686
CCK1R(Y140A) Control + Chol	7.4±0.2 (4) 7.7±0.1 (4)	0.057	0.3±0.1 (4) 0.3±0.1 (4)	0.886	8.1±0.3 (4) 8.4±0.3 (4)	0.200
CHO-CCK1R SRD15-CCK1R WT SRD15-CCK1R WT + MβCD	7.7±0.1 (4) 7.5±0.1 (6)	0.476	0.6±0.1 (4) 0.5±0.1 (6)	0.257	8.0±0.1 (4) 9.0±0.1 (5)*] * 7.8±0.4 (4)	0.016 0.016
CCK2R WT Control + Chol	8.4±0.1 (4) 8.4±0.1 (4)	0.886	0.5±0.1 (4) 0.5±0.1 (4)	0.886	10.4±0.1 (4) 10.2±0.2 (4)	0.486
CCK2R(sterol 7M) Control + Chol	8.2±0.1 (4) 8.1±0.1 (4)	0.486	0.5±0.1 (4) 0.4±0.1 (4)	0.200	9.6±0.2 (4) 9.7±0.4 (4)	0.886
***CCK-4***						
CCK1R WT Control + Chol	4.2±0.2 (5) 4.0±0.5 (5)	0.841	-	-	5.5±0.1 (9) 6.2±0.1 (9)*	0.001
CCK1R(sterol 7M) Control + Chol	<4.0 <4.0	-	-	-	6.7±0.2 (7) 6.8±0.2 (7)	0.620
CCK1R(Y140A) Control + Chol	4.3±0.2 (6) 4.3±0.2 (6)	0.937	0.1±0.1 (6) 0.1±0.1 (6)	0.792	5.7±0.1 (8) 5.7±0.1 (8)	0.982
CHO-CCK1R SRD15-CCK1R WT SRD15 CCK1R WT + MβCD	4.5±0.1 (4) 5.5±0.2 (4)*	0.029	0.1±0.1 (4) 0.2±0.1* (4)	0.029	5.3±0.1 (8) 6.8±0.1 (8)* ]* 5.7±0.2 (7)	0.0002 0.0003
CCK2R WT Control + Chol	7.7±0.1 (6) 7.8±0.1 (6)	0.818	0.6±0.1 (6) 0.5±0.1 (6)	0.240	9.5±0.2 (8) 9.4±0.2 (8)	0.879
CCK2R(sterol 7M) Control + Chol	7.2±0.1 (6) 7.6±0.1 (6)	0.095	0.6±0.1 (6) 0.5±0.1 (6)	0.699	9.1±0.2 (7) 9.2±0.1 (7)	0.620

Values are expressed as mean ± SEM from duplicate determinations in “n” independent experiments (replicate number in parentheses). Data were analyzed using one-way ANOVA with Tukey’s multiple comparison test.

* *p* < 0.05, significantly different from the control for the same cell line. Underlying data are found in S1 Data.

In summary, CCK1R is sensitive to elevated membrane cholesterol, disrupting its normal stimulus-response coupling primarily by stabilizing Gq/11 interaction leading to a reduced CCK-8 dissociation rate, higher apparent binding affinity, and lower potency for intracellular calcium signaling. In contrast, CCK2R is insensitive to elevated cholesterol, with slower G protein activation and slow peptide off rate, even in a low cholesterol environment. These distinctions in receptor function are dependent upon differential cholesterol interaction with a specific CRAC motif in TM3 located in proximity to intracellular loop 2 of the receptor, a region that is known to be important in G protein activation. Since cholesterol disrupts normal stimulus-response coupling, it may be possible to correct this aberration via a positive allosteric modulator (PAM) that can act outside of the deep orthosteric binding pocket. Such a “corrective PAM” could reintroduce CCK1R to the list of possible targets for the treatment of obesity.

## Materials and methods

### Materials

Synthetic cholecystokinin (CCK) peptides were purchased from Bachem (Torrance, California) or prepared in our laboratory. We utilized the traditional nomenclature for these peptides based on the 33-residue natural porcine peptide first isolated and utilized in all early studies, representing CCK(1–33). The C-terminal octapeptide of this, CCK(26–33), is identified as CCK-8, the most common synthetic peptide utilized subsequently, with the tyrosyl residue in this peptide sulfated unless noted as CCK-8-desulfate (CCK-8-DS). Similarly, the C-terminal tetrapeptide, CCK(29–33), is identified as CCK-4. Hams F-12, Dulbecco’s modified Eagle’s medium (DMEM), DMEM-F12 mixture medium, soybean trypsin inhibitor, and zeosin were purchased from Invitrogen (Carlsbad, California). Radioactive iodine (Na^125^I) was from Perkin-Elmer (Boston, Massachusetts). The fluorescent CCK analogs, alexa^488^-Gly-[(Nle^28,31^)CCK-26-33] [24] and Gly-[(Nle^28,31^)CCK-26-32]-aladan^33^ [34] and photoaffinity labeling probe, (des-amino-Tyr)-Gly-[(Nle^28,31^)CCK(26–32)]-Bpa^33^ [28] were synthesized in our laboratory with identity confirmed by mass spectrometry. Fetal clone II and all tissue culture supplements were from Life Technologies (Carlsbad, California). Cyanogen bromide (CNBr) was purchased from Pierce Chemicals (Rockford, Illinois). Lipoprotein-deficient serum was from Intracel (Frederick, Maryland). Quest Fluo-8-AM was from AAT Bioquest Inc. (Sunnyvale, California). Cholesterol, 25-hydroxy-cholesterol, methyl-β-cyclodextrin (MβCD), probenecid and guanosine 5′-[β,γ-imido]triphosphate trisodium salt (GppNHp) were from Sigma-Aldrich (St. Louis, Missouri). All other reagents were of analytical grade.

### Cell lines

CHO-K1 cells expressing WT and mutant (CCK1R(Y140A), CCK1R(Y140T), and CCK1R(Y140F)) CCK receptors were prepared by transfecting non-receptor-bearing CHO cells using PEI method [10]. We also prepared stable HEK-293 cell lines expressing WT CCK1R, WT CCK2R, CCK1R(Y140A), CCK1R(sterol 7M), and CCK2R(sterol 7M) constructs, as well as similar constructs in HEK-293 cells without Gq/11. These cells were prepared using the CRISPR-Cas9 approach by Dr. Asuka Inoue (Tohoku University, Miyagi, Japan) [27]. Clonal cell lines were isolated by limiting dilution using either G-418 or zeosin (1/0.5 mg/ml) selection and cells with similar levels of receptor expression were used for this study. Receptor expression levels were determined by CCK radioligand binding assay [10]. Cells were grown either in Ham-F12 medium supplemented with 5% Fetal Clone II or DMEM supplemented with 10% Fetal Clone II with 1% penicillin-streptomycin, 4 mM glutamine mixture in a humidified atmosphere containing 5% CO_2_ at 37°C. SRD15 cells expressing CCK receptors were prepared and cultured as described previously [10]. For specific assays, receptor expressing KO Gq/11 cells were transfected with either Gαq or Gα11 by PEI method, and receptor-enriched membranes were prepared by discontinuous sucrose gradient centrifugation, as described previously [24].

### Receptor constructs and mutagenesis

CCK receptor constructs were prepared by the QuikChange site-directed mutagenesis kit (Agilent Technologies, Santa Clara, California). Sequences were confirmed by DNA sequencing.

#### Modification of membrane cholesterol levels

Cholesterol levels in receptor-bearing cells were increased using freshly prepared methyl-β-cyclodextrin(MβCD)-cholesterol complex according to Pontier and colleagues [39]. In brief, 180 mM cholesterol in 2-propanol was added dropwise to an aqueous solution of methyl-β-cyclodextrin (100 mg/1 ml) at 60 to 80°C with continuous shaking to yield a 1/10 MβCD-cholesterol ratio. Cells had their membrane cholesterol enhanced by incubation with 5 mM MßCD-cholesterol complex for 30 min at 37°C.

#### Radioiodination

Radioligands, (^125^I-des-amino-Tyr)-Gly-[(Nle^28,31^)CCK(26–33)] and (^125^I-des-amino-Tyr)-Gly-[(Nle^28,31^)CCK(26–32)]-Bpa^33^, were prepared by mixing 20 μg of peptide with 1 mCi of Na^125^I in the presence of iodination beads (Pierce Chemicals, Rockford, Illinois) for 15 s, and the products were purified by reversed-phase HPLC, yielding approximate specific radioactivities of 2,000 Ci/mmol [40].

#### Radioligand binding assay

Competition-binding assays were carried out in intact cells with and without cholesterol modification, as described previously [10]. Cells were seeded in 24-well clear tissue culture plates and allowed to grow to approximate 80% confluence. On the day of assay, cellular cholesterol content was enhanced as described above and cells were mixed with approximately 11.2 pM of (^125^I-des-amino-Tyr)-Gly-[(Nle^28,31^)CCK(26–33)] for 1 h in Kreb’s-Ringers-HEPES (KRH) medium containing 25 mM HEPES, pH 7.4, 104 mM NaCl, 5 mM KCl, 2 mM CaCl_2_, 1 mM KH_2_PO_4_, and 1.2 mM MgSO_4_, with 0.01% soybean trypsin inhibitor and 0.2% bovine serum albumin in the absence or presence of increasing concentrations (0 to 1 μM) of unlabeled CCK peptide analogs at room temperature. After incubation, the contents were aspirated and washed twice using ice-cold KRH medium, pH 7.4, to separate free from bound radioligand. Cells were lysed by vigorous shaking using 0.5 N NaOH and radioactivity was quantified using a LB2111 multicrystal γ-spectrometer. Non-specific binding in the presence of 1 μM unlabeled CCK-8 peptide was less than 20%. The assay was performed in duplicate, and the saturable binding was plotted by nonlinear regression curve fitting using Prism 9.2 (Graph Pad, San Diego, California). Data were fit to one- and two-site models, with F-test determining if the two-site model was significantly better than the one-site model, with *p* < 0.05 considered significant. Two-site data were utilized only when the F-test was significant.

#### Intracellular calcium assay

CCK-stimulated biological responses were determined by measuring intracellular calcium responses. In brief, cells were seeded at a density of approximately 20,000 cells/well on a poly-lysine coated black clear-bottom 96-well plates. Cells were washed with calcium assay buffer (25 mM HEPES (pH 7.4), 104 mM NaCl, 5 mM KCl, 1.5 mM CaCl_2_, 1.0 mM KH_2_PO_4_, 1.2 mM MgSO_4_, 1.2 mM MgCl_2_, 0.2% bovine serum albumin, 2.5 mM probenecid) and assays were initiated after cholesterol modification as described above and after loading with 0.75 μM Fluo-8-AM for 45 min at 37°C in the dark. Assays were performed by robotic addition of increasing concentrations of agonist ligand (0 to 100 μM) using a FlexStation 3.0 plate reader equipped with Softmax Pro 5.4 software (Molecular Devices, Sunnyvale, California). Intracellular calcium responses were determined by measuring the fluorescence emission intensity at 525 nm after excitation at 485 nm, with data collected every 4 s for 120 s. All assays were performed in duplicate and repeated in a minimum of 3 independent experiments. The peak calcium responses were analyzed and plotted as percentages of the maximal response to 0.1 mM ATP using nonlinear regression analysis in the Prism 9.2.

#### Cryo-EM structural studies

Receptor complex purifications: Protein complexes were generated and isolated in accordance with the protocol reported by Mobbs and colleagues [12] with minor modifications. High Five (*Trichoplusia ni*, Thermo Fisher Scientific) cells were maintained in suspension culture at 27°C using ESF-291 medium (Expression Systems). Recombinant baculoviruses were produced using the Bac-to-Bac system (Thermo Fisher Scientific). Cells (3 × 10^6^ cell/ml) were infected with baculoviruses and harvested by centrifugation after approximately 48 h of shaking at 27°C. Insect cell pellets were frozen and stored at −80°C until use.

For the CCK1R(sterol 7M)-containing complex, a High Five cell pellet (36 g from 1.25 L of culture) co-expressing a Gq protein-mimic construct fused to the C terminus of the mutant CCK1R [12], Gβ1, and Gγ2 was thawed and resuspended in hypotonic lysis buffer (125 ml, 20 mM HEPES, 5 mM MgCl_2_, 1 μM CCK-8, 2 × EDTA-free protease inhibitor tablets (Roche), pH 7.4). The mixture was stirred for 15 min at room temperature before centrifugation (20,000 × g, 15 min, 4°C). The supernatant was discarded, and the pellet was resuspended in buffer (150 ml, 20 mM HEPES, 100 mM NaCl, 5 mM MgCl_2_, 5 mM CaCl_2,_ 5 μM CCK-8, 2 × EDTA-free protease inhibitor tablets, pH 7.4). The resulting mixture was supplemented with ScFv16 [41] (227 μg, Invitrogen), apyrase (5 μl, New England Biolabs), and benzonase (1 μl, Millipore Sigma). A solution of detergent (20 ml, 5% LMNG, 0.3% CHS, in ultrapure water) was slowly added to the mixture while stirring. The resulting mixture was Dounce homogenized, 80 ml buffer (20 mM HEPES, 100 mM NaCl, and 5 mM MgCl_2_) was added, and NaCl (5 M stock in H_2_O) and CaCl_2_ (1 M stock in H_2_O) were added to maintain NaCl and CaCl_2_ concentrations of 100 mM and 5 mM, respectively. After stirring at 4°C for 1 h, the mixture was centrifuged (6,000×g, 20 min, 4°C), and filtered with a glass fiber prefilter. M1 anti-FLAG resin (approximately 3 ml) was added to the filtrate, and batch binding was performed by rotating for 2 h at room temperature. The anti-FLAG resin was loaded onto a glass-fritted column and washed with 150 ml of ice-cold wash buffer (20 mM HEPES, 100 mM NaCl, 5 mM MgCl_2_, 5 mM CaCl_2_, 1 μM CCK-8, 0.01% LMNG, and 0.0006% CHS). Protein was eluted from the resin with 15 ml of elution buffer (20 mM HEPES, 100 mM NaCl, 5 mM MgCl_2_, 10 mM EGTA, 0.2 mg/ml FLAG peptide, 1 μM CCK-8, 0.01% LMNG, and 0.0006% CHS). An additional 250 μg of ScFv16 was added to the elution volume, mixed thoroughly, and the resulting solution was concentrated to approximately 0.6 ml with a centrifugal concentrator (100 kDa MWCO). The solution was spin-filtered (0.22 μm) and injected onto a Biorad system equipped with a GE Superdex 200 Increase 10/300 GL column, BioLogic Dual flow, BioFrac Fraction Collector, and a Shimadzu RF10AXL Fluorescence detector. The complex was resolved with a 0.5 ml/min flow of SEC buffer (20 mM HEPES, 100 mM NaCl, 5 mM MgCl_2,_ 2 μM CCK-8, 0.01% LMNG, and 0.0006% CHS, pH 7.4). Fractions (300 μl) containing the desired protein complex were pooled and concentrated to 7.8 mg/ml with a 50 kDa MWCO centrifugal concentrator. Aliquots of the concentrate were flash frozen in liquid nitrogen and stored at −80°C for later use.

The CCK1R(Y140A)-containing complex was purified in an identical manner as above, except a cell pellet (30 g) from 1 L of culture was used, and the volumes of the lysis and resuspension buffers were scaled accordingly. The lysis, resuspension, and SEC buffers contained 2 μM, 10 μM, and 1 μM of CCK-8, respectively. The pooled SEC fractions were concentrated to 5 mg/ml using a 100 kDa MWCO centrifugal concentrator.

Samples were prepared for SDS-PAGE as previously reported [42] with a 1:1:1 mixture of sample, 10% (wt/vol) SDS (aq.) and Laemmli loading buffer containing 2-mercaptoethanol. Sample mixtures were not heated before loading onto gels. SDS–PAGE samples (10 ml) were loaded and run on Mini-PROTEAN TGX Precast 4%–15% gels at 200V for 30 min. SDS–PAGE gels were stained with InstantBlue Coomassie stain (Abcam). Flash-frozen samples were thawed and stored briefly on ice. TEM grids (either UltrAuFoil r1.2/1.3 or Au-coated [43] Quantifoil r1.2/1.3) were glow discharged using a GloQube Plus instrument (air chamber, 20 mA, 60 s or 150 s, negative polarity). The grids were loaded into a Vitrobot MkIV (Thermo Fisher Scientific, 4°C, 100% humidity), sample (3 μl) was pipetted onto the grids, the grids were blotted, and plunge frozen in liquid ethane. Specific parameters for grid preparation can be found in S2 and S3 Figs.

Cryo-EM grids were clipped under liquid nitrogen and screened using a Thermo Fisher Scientific Glacios (200 kV) microscope equipped with a Falcon 4 camera. Selected grids were transferred to a Titan Krios G1 microscope equipped with Gatan K3 camera and Gatan energy filter. The instrument was operated at an accelerating voltage of 300 kV and with an indicated magnification of 105,000X, resulting in a pixel size of 0.85 Å. Automated data acquisition was performed using EPU 2 software (Thermo Fisher Scientific) with aberration free image shift (AFIS). The energy filter was operated with a slit-width of 10 eV, the camera was operated in correlated double sampling (CDS) mode, and movies were recorded as multi-frame (60 subframes) TIFF files. The total dosage applied to the samples was 60 e^-^/Å^2^. The dosage rates were 8.9 e/px/s and 8.56 e/px/s for the CCK1R(sterol 7M) and CCK1R(Y140A) containing samples, respectively. The number of movies recorded were 6878 and 5884 for the CCK1R(sterol 7M)- and CCK1R(Y140A)-containing samples, respectively.

TIFF files were pre-processed using the EPU_Group_AFIS.py script (https://github.com/DustinMorado/EPU_group_AFIS) for import into RELION 3.1.2 [44]. MotionCor2 as implemented in RELION 3.1.2 was used for patch motion correction [45]. Contrast transfer function (CTF) estimation was performed with CTFFIND 4.1.14 [46] and micrographs were selected by estimated maximum resolution. Particle picking was performed with crYOLO 1.7.6 [47]. Multiple rounds of 2D-classification and ab initio reconstruction were performed with cryoSPARC 4.1.2 [48]. Bayesian polishing was performed with RELION 3.1.2 [49]. Refinement with defocus and CTF refinement was performed with cryoSPARC 4.1.2 3D-classification and 3D-variability analysis [50] were performed using cryoSPARC 4.1.2. Details for processing can be found in S2 and S3 Figs. The structure of the CCK-8/CCK1R-mGsqi complex (PDB: 7MBY [12]) was used as a starting model and loaded into ChimeraX 1.3 [51]. Initial flexible fitting into cryo-EM maps was performed using ISOLDE 1.3 [52]. Manual adjustments were performed using COOT 0.9.6 [53] and real-space refinement and validation were performed using PHENIX 1.19.2 [54].

#### Fluorescence collisional quenching experiments

Fluorescence collisional quenching studies were performed as described previously [34]. Samples were prepared by mixing the receptor-enriched membrane suspension (10 μg) with 50 nM Gly-[(Nle^28,31^)CCK-26-32]-aladan^33^ probe in the absence or presence of 10 μM GppNHp for 30 min at room temperature in KRH medium, pH 7.4. After incubation, the reactions were terminated by adding cold buffer and the receptor-bound fractions were separated from free ligand by centrifugation at 25,000×g for 5 min at 4°C. Cell pellets were washed again with ice-cold KRH medium and resuspended in KRH medium for fluorescence measurements. Fluorescence intensities were collected by exciting samples at 362 nm and emission intensities were collected at 505 nm using Fluoromax-3 spectrofluorometer equipped with Origin version 8.1. Fluorescence signal was collected after sequential addition of potassium iodide (KI), a hydrophilic quencher (1M KI aqueous solution in 10 mM Na_2_S_2_O_3_) with an integration time of 10 s, with 3 repetitions for each value. Background-subtracted corrected fluorescence data were calculated and plotted based on the equation, F_o_/F = 1+Ksv[Q], where F_o_/F is the fluorescence intensity in the presence or absence of KI. The quenching constant, K_SV_, was calculated from the slope of F_o_/F as a function of the quencher concentration [I^-^].

#### Fluorescence polarization kinetic assay

Fluorescence polarization assays were performed using receptor-enriched membranes following the fluorescence anisotropy protocol (Ex 482 nm, Em 522 nm), with measurements read for 0.5 s/cycle for a total of 200 flashes using PherastarFSX plate reader [8]. Ligand binding was initiated by mixing receptor-enriched membranes (4 μg/mg protein) with alexa^488^-Gly-[(Nle^28,31^)CCK-26-33] probe (3.2 nM or 10 nM) in a final volume of 200 μl KRH medium (pH 7.4) with 0.2% bovine serum albumin for a total of 75 cycles. This was done in the absence or presence of the non-hydrolyzable GTP analogue, GppNHp, at 10 μM concentration. Association rate was collected for 50 cycles to reach plateau signal then dissociation of CCK probe was initiated by adding 1 μM unlabeled CCK peptide and continuing to collect the signal for another 25 cycles. Assays were performed in triplicate and the specific signals were calculated by subtracting nonspecific signal (signal in the presence of unlabeled CCK) from the total signal. The final kinetic data were calculated using nonlinear regression curve fitting with association and then dissociation parameters with maximum iterations of fitting using Prism 9.2.

#### Photoaffinity labeling of CCK receptor

Receptor-enriched membranes (approximately 50 μg) were incubated with approximately 0.1 nM (^125^I-des-amino-Tyr)-Gly-[(Nle^28,31^)CCK(26–32)]-Bpa^33^ in 250 μl of KRH medium (pH 7.4) at 25°C for 60 min in the dark. After incubation, contents were transferred to a siliconized glass tube and exposed to UV irradiation for 30 min at 4°C with 3,500-Å lamps in a Rayonet photochemical reactor (Southern New England Ultraviolet, Hamden, Connecticut). After photolysis, membrane suspensions were centrifuged at 25,000×g for 5 min and washed twice with ice-cold medium and then resuspended in 30 μl of SDS sample buffer prior to separation on 10% SDS-polyacrylamide gels. Gels were dried and radiolabeled bands were visualized by autoradiography. The apparent molecular weight of radiolabeled receptor fragments was determined from the mobility of Prosieve color protein standards (Cambrex, Rockland, Maine). Receptor radiolabeled bands were excised from the polyacrylamide gel, eluted, and lyophilized, then precipitated with ethanol. Radiochemically pure receptor suspension was cleaved with 2.5 mg of CNBr in 70% formic acid, as described previously [28]. The cleaved receptor products were washed, dried, and resolve in 10% NuPAGE gel with MES running buffer (Invitrogen, Carlsbad, California). The labeled products were identified by autoradiography.

#### Membrane BRET assay for G protein conformational changes

HEK293 cells with KO Gq/11 that stably expressed different CCK receptor constructs were transiently transfected with Gαq-Nluc, Gβ1 and Gγ2-Venus at a ratio of 1:1:1 for 24 h. Cell membranes were prepared as described previously [26]. Briefly, cells were harvested and suspended in membrane preparation buffer (20 mM BisTris (pH 7.4), 50 mM NaCl, 1 mM MgCl_2_, 1 × P8340 (protease inhibitor cocktail, Sigma), 1 mM DTT, and 0.1 mM PMSF), homogenized, applied to sucrose gradient (homogenate, 40%, 60%), and centrifuged at 100,000×g for 2.5 h at 4°C. The layer between the homogenate and 40% sucrose was collected and diluted in membrane buffer and centrifuged at 100,000×g for 20 min at 4°C. The final pellet, containing plasma membranes were resuspended in membrane preparation buffer, aliquoted, and stored at −80°C.

To perform G protein conformation assays, 5 μg per well of cell membrane was incubated with furimazine (1:1,000 dilution from stock) in assay buffer (1× HBSS, 10 mM HEPES, 0.1% (w/v) ovalbumin, 1 mM DTT and 0.1 mM PMSF, 1 × P8340, pH 7.4). Ligand-mediated change in BRET between Gα and Gγ was measured at 30°C using a PHERAstar (BMG LabTech with Emission 1: 475/30 nm and Emission 2: 535/30 nm). Baseline BRET measurements were taken for 2 min before addition of vehicle or increasing concentrations of CCK-8. BRET was measured at 15 s intervals for a further 10 min. A saturating concentration of GTP (30 μM) was then added to induce subunit dissociation. The BRET signal was calculated as the ratio of the 535/30 nm emission over the 475/30 nm emission. Data were baseline and vehicle corrected. Concentration-response curves to evaluate changes in G protein conformation were constructed using area-under-the-curve from the data collected from 0 to 10 min following ligand addition, prior to the addition of GTP.

#### TRUPATH assay of G protein activation

HEK293 cells with KO Gq/11 that stably expressed different CCK receptor constructs were transfected with TRUPATH sensor including Gαq-Rluc8 or Gα11-Rluc8, with Gβ3 and Gγ9-GFP2 at 1:1:1 ratio using PEI. Cells were then seeded into white 96-well dishes at a density of 30,000 cells/well for 48 h. Cell culture media was then replaced with assay buffer (1 × HBSS, 10 mM Hepes (pH 7.4), with 0.1% ovalbumin). Cells were incubated in assay buffer for 30 min at 37°C. Prolume purple coelenterazine (Nanolight Technologies, Pinetop, Arizona) was then added to the plate at a final concentration of 1.3 μM and incubated for a further 10 min at 37°C. BRET measurements were performed on a PHERAstar plate reader (BMG Labtech) using BRET2 optics (410/80 nm/515/30 nm), with baseline measurements taken for 10 min before addition of vehicle or peptide and reading for a further 20 min. BRET signal was calculated as the ratio of the 515/30 nm emission over the 410/80 nm emission. This ratio was vehicle and baseline-corrected. For a subset of experiments, membrane cholesterol enrichment was performed as described previously.

#### Statistics

Comparisons between experimental conditions were evaluated by either nonparametric unpaired *t* test with Mann–Whitney post-test or by one-way analysis of variance (ANOVA) with Tukey’s or Dunnett’s multiple comparison test. Values of *p* < 0.05 were considered to be statistically significant.

## Supporting information

S1 Fig Purification and characterization of CCK1R-complexes containing (sterol 7M) mutations (F130L, S136A, G141S, I216L, L219F, I223V, M226A) (A, B) and the (Y140A) mutation (C, D). Preparative SEC chromatograms (A, C) and SDS-PAGE samples stained with Coomassie (B, D). Sol. indicates solubilized fraction, F.T. indicates anti-flag column flow through, and elute indicates anti-FLAG column elution. Post-SEC indicates samples that were purified by size-exclusion chromatography. Fractions were pooled with retention times indicated by the dotted lines on the SEC chromatograms. For the CCK1R(sterol 7M)-containing complex, the sample labeled post SEC 2 was used for cryo-EM studies, and for the CCK1R(Y140A)-containing complex, the sample labeled post-SEC 1 was used for cryo-EM studies. Uncropped images can be found in S1 Raw Images.
(TIF)

S2 Fig (A) Cryo-EM sample preparation and processing pipeline for the CCK1R(sterol 7M)-containing complex. The field of view for the representative micrograph is 490 nm by 348 nm. (B) A map with a local resolution estimation shown by color. (C) A Fourier shell correlation (FSC) plot for the map shown in subpanel B. (D) A particle distribution histogram from the reconstruction shown in subpanel B. (E) Map-to-model FSC plot. The mask for the masked FSC curve was generated by PHENIX 1.19.2. Dotted lines indicate FSC values of 0.143 and 0.5, which correspond to values of 2.43 Å and 2.83 Å, respectively, for the masked FSC curve and 2.47 Å and 2.95 Å, respectively, for the unmasked FSC curve.
(TIF)

S3 Fig (A) Cryo-EM sample preparation and processing pipeline for the CCK1R(Y140A)-containing complex. The field of view for the representative micrograph is 490 nm by 348 nm. (B) A map with a local resolution estimation shown by color. (C) A Fourier shell correlation (FSC) plot for the map shown in subpanel B. (D) A particle distribution histogram from the reconstruction shown in subpanel B. (E) Map-to-model FSC plot. The mask for the masked FSC curve was generated by PHENIX 1.19.2. Dotted lines indicate FSC values of 0.143 and 0.5, which correspond to values of 2.61 Å and 3.12 Å, respectively, for the masked FSC curve and 2.72 Å and 3.23 Å, respectively, for the unmasked FSC curve.
(TIF)

S4 Fig Cryo-EM imaging, processing, and model statistics. (TIF)

S1 Data Numerical data for figures. Excel spreadsheet containing, in separate sheets, the underlying numerical data for Figs 1, 2, 4, 6, 7A–7D, 7E–7G, 8A, 8B, 9 (top row), and 9 (bottom row).
(XLSX)

S1 Raw Images Original images for blots and gels. This includes uncropped images included in Figs 5 and S1.
(PDF)

## Abbreviations

AFIS: aberration free image shift
CDS: correlated double sampling
CTF: contrast transfer function
DMEM: Dulbecco’s modified Eagle’s medium
FP: fluorescence polarization
GPCR: G protein-coupled receptor
PAM: positive allosteric modulator
WT: wild-type
