# Supplementary figures and images for: Cholesterol-dependent dynamic changes in the conformation of the type 1 cholecystokinin receptor affect ligand binding and G protein coupling
Source: PLoS Biol. 2024 Jul 31;22(7):e3002673. doi: 10.1371/journal.pbio.3002673 (PMC11290853; doi:10.1371/journal.pbio.3002673)

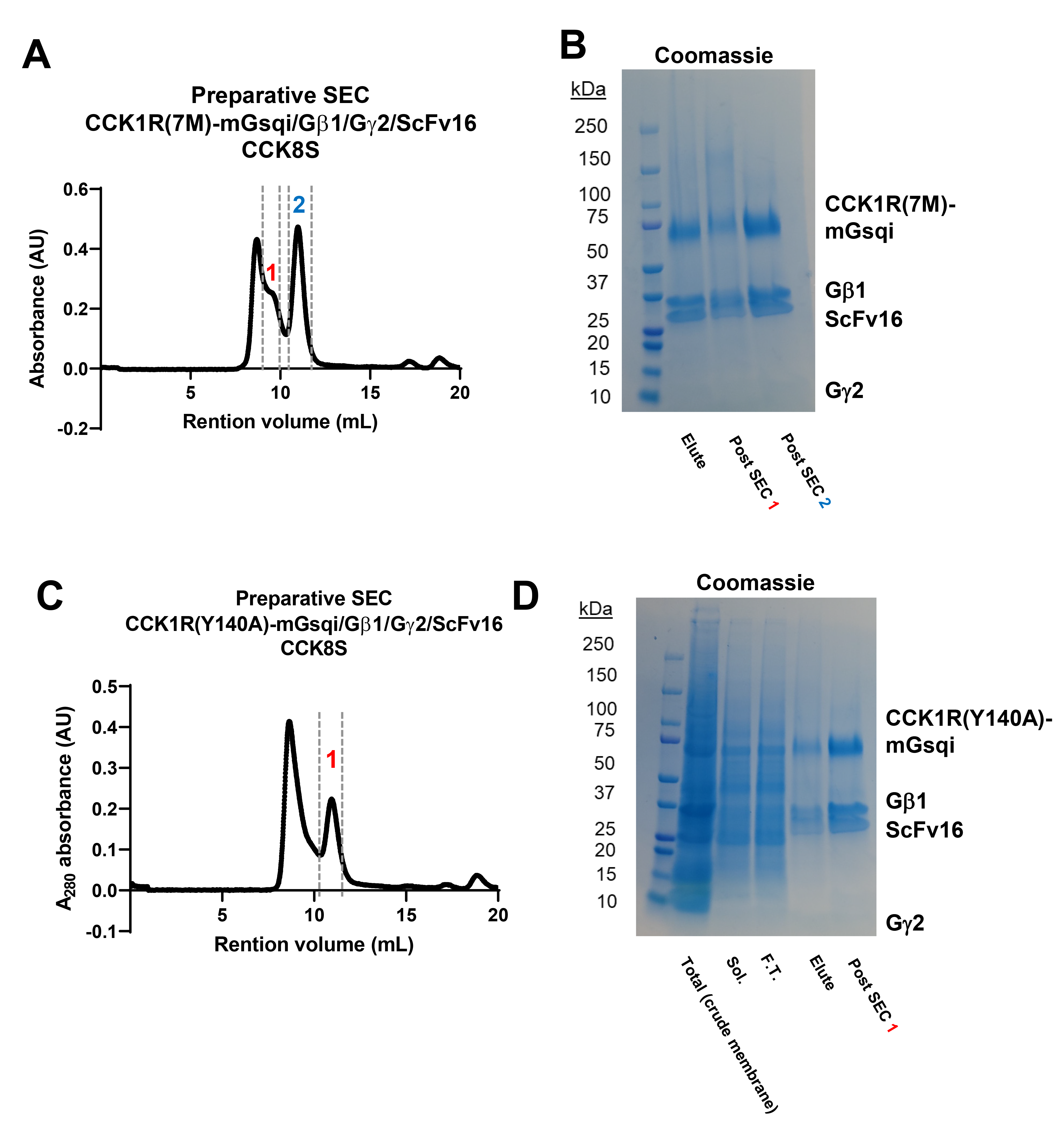

Supplement: S1 Fig — Purification and characterization of CCK1R-complexes containing (sterol 7M) mutations (F130L, S136A, G141S, I216L, L219F, I223V, M226A) (A, B) and the (Y140A) mutation (C, D). Preparative SEC chromatograms (A, C) and SDS-PAGE samples stained with Coomassie (B, D). Sol. indicates solubilized fraction, F.T. indicates anti-flag column flow through, and elute indicates anti-FLAG column elution. Post-SEC indicates samples that were purified by size-exclusion chromatography. Fractions were pooled with retention times indicated by the dotted lines on the SEC chromatograms. For the CCK1R(sterol 7M)-containing complex, the sample labeled post SEC 2 was used for cryo-EM studies, and for the CCK1R(Y140A)-containing complex, the sample labeled post-SEC 1 was used for cryo-EM studies. Uncropped images can be found in S1 Raw Images. (TIF) [file pbio.3002673.s001.tif]

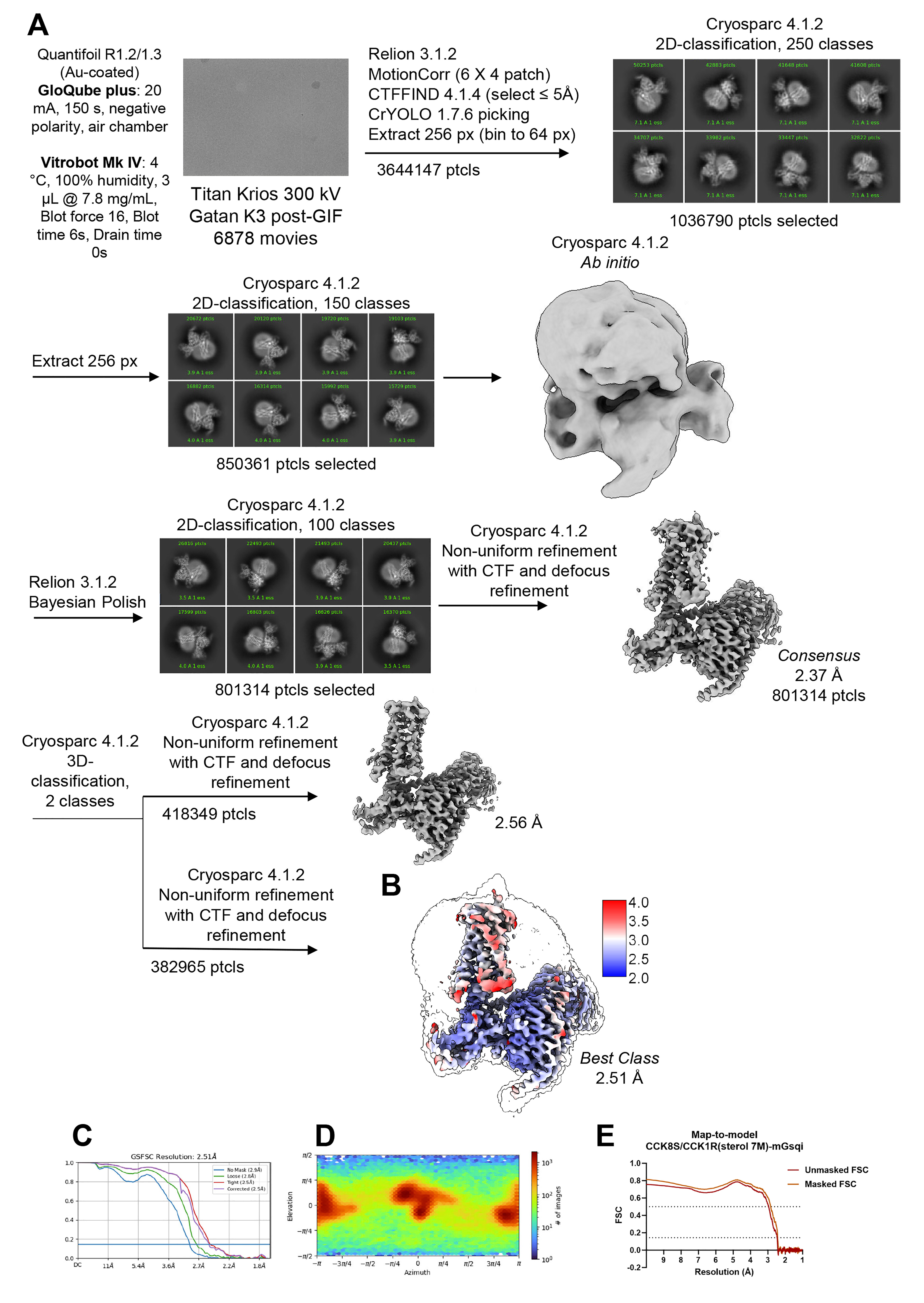

Supplement: S2 Fig — (A) Cryo-EM sample preparation and processing pipeline for the CCK1R(sterol 7M)-containing complex. The field of view for the representative micrograph is 490 nm by 348 nm. (B) A map with a local resolution estimation shown by color. (C) A Fourier shell correlation (FSC) plot for the map shown in subpanel B. (D) A particle distribution histogram from the reconstruction shown in subpanel B. (E) Map-to-model FSC plot. The mask for the masked FSC curve was generated by PHENIX 1.19.2. Dotted lines indicate FSC values of 0.143 and 0.5, which correspond to values of 2.43 Å and 2.83 Å, respectively, for the masked FSC curve and 2.47 Å and 2.95 Å, respectively, for the unmasked FSC curve. (TIF) [file pbio.3002673.s002.tif]

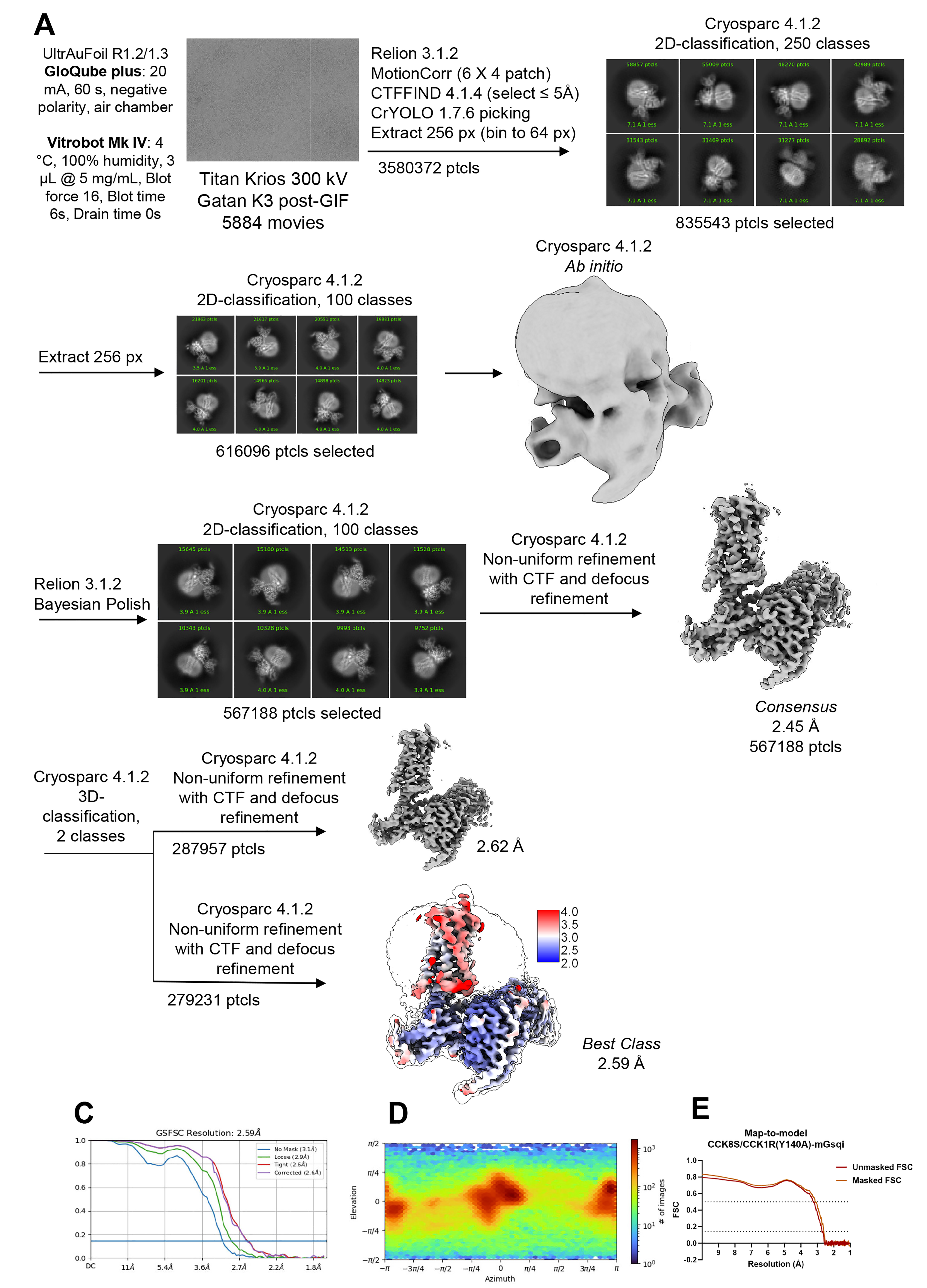

Supplement: S3 Fig — (A) Cryo-EM sample preparation and processing pipeline for the CCK1R(Y140A)-containing complex. The field of view for the representative micrograph is 490 nm by 348 nm. (B) A map with a local resolution estimation shown by color. (C) A Fourier shell correlation (FSC) plot for the map shown in subpanel B. (D) A particle distribution histogram from the reconstruction shown in subpanel B. (E) Map-to-model FSC plot. The mask for the masked FSC curve was generated by PHENIX 1.19.2. Dotted lines indicate FSC values of 0.143 and 0.5, which correspond to values of 2.61 Å and 3.12 Å, respectively, for the masked FSC curve and 2.72 Å and 3.23 Å, respectively, for the unmasked FSC curve. (TIF) [file pbio.3002673.s003.tif]

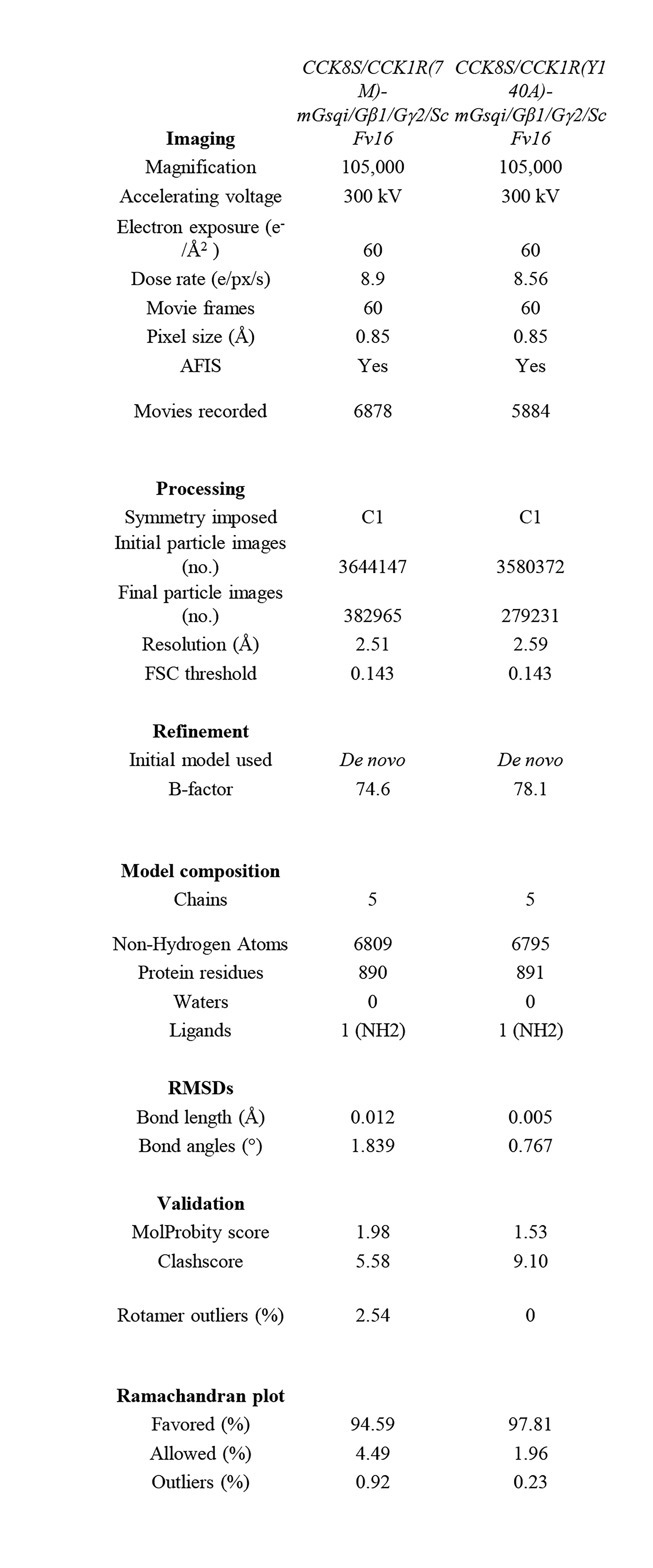

Supplement: S4 Fig — (TIF) [file pbio.3002673.s004.tif]
