# Supplementary material for: Cholesterol-dependent dynamic changes in the conformation of the type 1 cholecystokinin receptor affect ligand binding and G protein coupling
Source: PLoS Biol. 2024 Jul 31;22(7):e3002673. doi: 10.1371/journal.pbio.3002673 (PMC11290853; doi:10.1371/journal.pbio.3002673)
Supplement: S1 Raw Images — This includes uncropped images included in Figs 5 and S1. (PDF) [file pbio.3002673.s006.pdf]

Fig 5. Photoaffinity labeling of CCK receptor constructs.

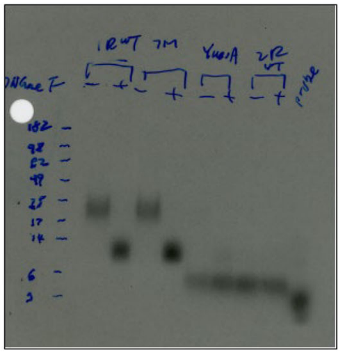

Autoradiograph of 10% NuPAGE gel

Lane 1 - Molecular weight markers: 182, 98, 62, 49, 28, 17, 14, 6, 3 kDa
Lane 2 - CNBr cleaved CCK1R WT
Lane 3 - CNBr cleaved CCK1R WT, PNGase F treated
Lane 4 - CNBr cleaved CCK1R(sterol 7M)
Lane 5 - CNBr cleaved CCK1R(sterol 7M), PNGase F treated
Lane 6 - CNBr cleaved CCK1R(Y140A)
Lane 7 - CNBr cleaved CCK1R(Y140A), PNGase F treated
Lane 8 - CNBr cleaved CCK2R WT
Lane 9 - CNBr cleaved CCK2R WT, PNGase F treated
Lane 10 - probe: (^{125}I-des-amino-Tyr)-Gly-[(Nle^{28,31})CCK(26-32)]-Bpa^{33}

S1 Fig. Purification of CCK1R complexes.

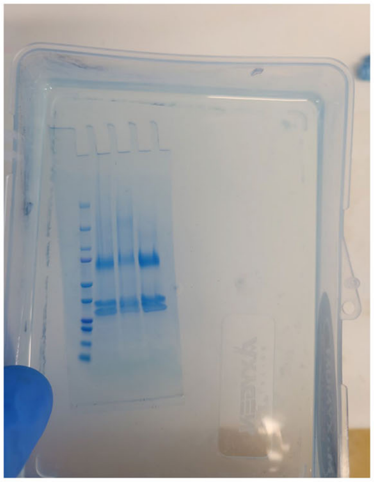

Panel B. CCK1R(7M)−mGsqi purification

Lane 1 - Molecular weight markers: 250, 150, 100, 75, 50, 37, 25, 20, 15, 10 kDa
Lane 2 - Eluate from anti-flag resin
Lane 3 - Material purified on size exclusion chromatography, fraction #1
Lane 4 - Material purified on size exclusion chromatography, fraction #2. This fraction was used for Cryo EM

S1 Fig. Purification of CCK1R complexes.

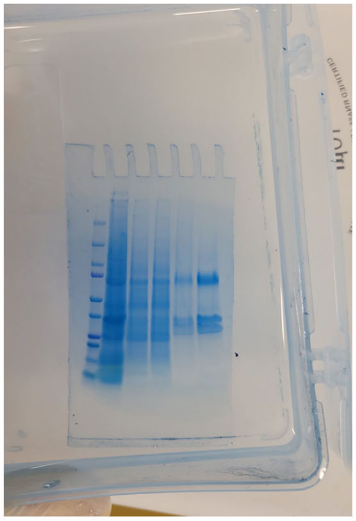

Panel D. CCK1R(Y140A)−mGsqi purification

Lane 1 - Molecular weight markers: 250, 150, 100, 75, 50, 37, 25, 20, 15, 10 kDa
Lane 2 - Total crude membranes
Lane 3 - Solubilized fraction
Lane 4 - Flow through from the anti-flag resin column
Lane 5 - Eluate from anti-flag resin column
Lane 6 - Material purified on size exclusion chromatography, fraction #1. This fraction was used for Cryo EM
